# Antimicrobial and Cell-Friendly Properties of Cobalt and Nickel-Doped Tricalcium Phosphate Ceramics

**DOI:** 10.3390/biomimetics9010014

**Published:** 2023-12-31

**Authors:** Dina V. Deyneko, Vladimir N. Lebedev, Katia Barbaro, Vladimir V. Titkov, Bogdan I. Lazoryak, Inna V. Fadeeva, Alevtina N. Gosteva, Irina L. Udyanskaya, Sergey M. Aksenov, Julietta V. Rau

**Affiliations:** 1 Chemistry Department, Lomonosov Moscow State University, Leninskie Gory 1, 119991 Moscow, Russia; vladimir.lebedev@chemistry.msu.ru (V.N.L.); vlatitkov@yandex.ru (V.V.T.); bilazoryak@gmail.com (B.I.L.); 2 Laboratory of Arctic Mineralogy and Material Sciences, Kola Science Centre RAS, 14 Fersman Str., 184209 Apatity, Russia; aks.crys@gmail.com; 3 Istituto Zooprofilattico Sperimentale Lazio e Toscana “M. Aleandri”, Via Appia Nuova 1411, 00178 Rome, Italy; katia.barbaro@izslt.it; 4 A.A. Baikov Institute of Metallurgy and Material Science, Russian Academy of Sciences, Leninsky Prospect 49, 119334 Moscow, Russia; fadeeva_inna@mail.ru; 5 Tananaev Institute of Chemistry, Kola Science Centre RAS, Akademgorodok 26A, 184209 Apatity, Russia; angosteva@list.ru; 6 Department of Analytical, Physical and Colloid Chemistry, Institute of Pharmacy, I.M. Sechenov First Moscow State Medical University, Trubetskaya 8, Build. 2, 119048 Moscow, Russia; udyanskaya_i_l@staff.sechenov.ru; 7 Geological Institute, Kola Science Centre, Russian Academy of Sciences, 14 Fersman Street, 184209 Apatity, Russia; 8 Istituto di Struttura della Materia, Consiglio Nazionale delle Ricerche (ISM-CNR), Via del Fosso del Cavaliere 100, 00133 Rome, Italy

**Keywords:** TCP, substituted tricalcium phosphate, doped tricalcium phosphate, whitlockite, calcium phosphate, antimicrobial, antibacterial, infection, bone regeneration, bone implant, cobalt, nickel

## Abstract

β-Tricalcium phosphate (β-TCP) is widely used as bone implant material. It has been observed that doping the β-TCP structure with certain cations can help in combating bacteria and pathogenic microorganisms. Previous literature investigations have focused on tricalcium phosphate structures with silver, copper, zinc, and iron cations. However, there are limited studies available on the biological properties of β-TCP containing nickel and cobalt ions. In this work, Ca_10.5−*x*_Ni*_x_*(PO_4_)_7_ and Ca_10.5−*x*_Co*_x_*(PO_4_)_7_ solid solutions with the β-Ca_3_(PO_4_)_2_ structure were synthesized by a high-temperature solid-state reaction. Structural studies revealed the β-TCP structure becomes saturated at 9.5 mol/% for Co^2+^ or Ni^2+^ ions. Beyond this saturation point, Ni^2+^ and Co^2+^ ions form impurity phases after complete occupying of the octahedral M5 site. The incorporation of these ions into the β-TCP crystal structure delays the phase transition to the α-TCP phase and stabilizes the structure as the temperature increases. Biocompatibility tests conducted on adipose tissue-derived mesenchymal stem cells (aMSC) using the (3-[4,5-dimethylthiazol-2-yl]-2,5 diphenyl tetrazolium bromide) (MTT) assay showed that all prepared samples did not exhibit cytotoxic effects. Furthermore, there was no inhibition of cell differentiation into the osteogenic lineage. Antibacterial properties were studied on the *C. albicans* fungus and on *E. coli*, *E. faecalis*, *S. aureus,* and *P. aeruginosa* bacteria strains. The Ni- and Co-doped β-TCP series exhibited varying degrees of bacterial growth inhibition depending on the doping ion concentration and the specific bacteria strain or fungus. The combination of antibacterial activity and cell-friendly properties makes these phosphates promising candidates for anti-infection bone substitute materials.

## 1. Introduction

Low-temperature modification of tricalcium phosphate (β-Ca_3_(PO_4_)_2_, β-TCP) is used in medicine for the restoration of damaged bone tissue [1,2,3]. In the regenerative medicine approach, the graft not only replaces the missing tissue but also provides strength to the bone and stimulates the growth of new bone at the defect site. Ideally, the new bone should penetrate and replace the graft through successive cycles of remodeling, maintaining an optimal balance between form and function. While auto-transplantation is still considered the “gold standard” for bone volume, complications can arise at the donor site [4]. Additionally, the risk of bacterial infection during the postoperative period necessitates the use of antibiotics, which remains a challenging issue due to bacterial resistance and biofilm formation. Therefore, there is a need to develop new materials that not only have osteoconductive properties but also exhibit inhibition of the growth of common hospital bacteria strains and contribute to avoiding biofilm formation.

There are two main approaches to achieving antibacterial activity in materials: using antibiotic-loaded bone graft substitutes [5,6,7] or incorporating ions with antibacterial properties into the crystal structure of the bioactive calcium phosphates (CPs) [8,9,10] or composites [11,12,13,14]. In light of the pandemic emergency of antibiotic-resistant strains of bacteria, the latter method appears to be the most promising [15]. To enhance the antibacterial properties of β-TCP-based materials, a number of compositions were developed. These compositions contain ions with antibacterial properties, such as Zn^2+^ [16,17], Cu^2+^ [8,18], Mn^2+^ [19,20], Fe^3+^ [21,22,23], Ag^+^ [24], and Gd^3+^ ions [25,26,27,28], as well as borate or silicate anions [29,30].

Cobalt (Co^2+^) [31,32,33] and nickel (Ni^2+^) [34,35,36] ions also exhibit antibacterial activity against pathogenic bacterial strains. Moreover, the presence of Co^2+^ in the β-TCP ceramics can stimulate the osteogenic potential [37], significantly improve the angiogenic ability of the materials [38], and enhance vascularization in vivo [39]. These positive effects were associated with increased expression of vascular endothelial growth factor (VEGF) due to the hypoxic environment caused by Co^2+^-doping [40]. Due to their magnetic properties, Co^2+^-doped CPs can be used in a wide range of applications, such as targeted drug delivery, magnetic resonance imaging, and magnetic hyperthermia [31].

Ni^2+^ ions also have good ferromagnetic properties and remain stable under conditions of oxygen excess. These advantages make nickel ions suitable for use in MRI diagnostics [36]. It has been demonstrated that doping of CPs with Ni^2+^ can enhance the mechanical properties of ceramics [41] and affect the degree of crystallinity [42] and phase formation [43]. The literature data on physical and bioactive properties and optimum dopant concentrations of Ni^2+^- and Co^2+^-doped CPs are summarized in Table 1.

It should be stressed that determining the optimum concentration of doping ions is of great importance, as high concentrations in CPs can have negative consequences on bioactive properties. For instance, high concentrations of Co^2+^ or Ni^2+^ ions in CP materials have been found to suppress cell proliferation and osteogenic differentiation [48] and exhibit cytotoxicity [41].

In this study, our aim was to determine the optimum doping concentration of Co^2+^ or Ni^2+^ ions in the β-TCP-based ceramics and its impact on bioactive properties, including the viability of adipose tissue-derived mesenchymal stem cells (aMSC) and their osteogenic differentiation, along with their antibacterial effects. The powders with different mol.% doping concentrations of Ni^2+^ or Co^2+^ in the β-TCP structure were obtained using the solid-state method. The phase composition and the limit of the structure’s capacity in relation to Ni^2+^ or Co^2+^ ions were studied. The structures were refined using the Rietveld method, and the occupancy of the crystal sites was determined. The antibacterial properties of the prepared ceramic materials were tested on *E. coli*, *E. faecalis*, *S. aureus, P. aeruginosa* bacteria strains, and *C. albicans* fungus. The biocompatibility was investigated by the MTT assay on aMSC isolated from the lambs taken at the slaughterhouse. The differentiation of the aMSC into the osteogenic lineage was studied.

## 2. Materials and Methods

### 2.1. Synthetic Route

The series of nickel and cobalt-substituted tricalcium phosphates, Ca_10.5−*x*_Ni*_x_*(PO_4_)_7_ (abbreviated as Ni-TCP), with *x* = 0, 0.33, 0.67, 1.0, 1.33, 1.58, and Ca_10.5−*x*_Co*_x_*(PO_4_)_7_ (abbreviated as Co-TCP), with *x* = 0, 0.33, 0.5, 0.67, 1.0, were obtained through high-temperature solid-state synthesis. The doping concentrations and sample codes are listed in Table 2. Stoichiometric amounts of raw materials were used for the syntheses. The initial substances for synthesizing nickel- and cobalt-doped phosphates, such as CaHPO_4_⋅2H_2_O (99.9%), CaCO_3_ (99.9%), NiO (76.0%), or Co_3_O_4_ (99.9%), were purchased from Sigma-Aldrich (Gillingham, UK). Nickel oxide was pre-calcined at 600 °C for 12 h to remove nitrates. Cobalt oxide (CoO) was obtained by annealing Co_3_O_4_ at 950 °C for 12 h. These materials were then checked for phase purity using the powder X-ray diffraction method. Next, the starting materials were weighed according to the stoichiometry of the reaction and mixed in an agate mortar with the addition of acetone (Diaem, Moscow, Russia). The resulting mixtures were placed in corundum crucibles and heated in a muffle furnace (SNOL, Termopech, Istra, Moscow region, Russia). The synthesis was carried out in three stages. First, the samples were preheated at 500 °C and kept for 12 h. Then the samples were annealed at 900 °C and kept for 18 h with one intermediate grinding in an agate mortar. The formation of Ni^2+^- and Co^2+^-doped β-TCP was performed according to reactions (1) and (2):

14CaHPO_4_⋅2H_2_O + (7 − 2*x*)CaCO_3_ + 2*x*NiO → 2Ca_10.5−*x*_Ni*_x_*(PO_4_)_7_ + (7 − 2*x*)CO_2_↑ + 35H_2_O (1)

or

14CaHPO_4_⋅2H_2_O + (7 − 2*x*)CaCO_3_ + 2*x*CoO → 2Ca_10.5−*x*_Co*_x_*(PO_4_)_7_ + (7 − 2*x*)CO_2_↑ + 35H_2_O (2)

### 2.2. Characterization

#### 2.2.1. Powder X-ray Diffraction Study

The powder X-ray diffraction (PXRD) patterns of prepared phases and initial reagents were checked using the JCPDS PDF-2 database (International Centre for Diffraction Data, PA, USA). The heating treatment temperature was chosen in accordance with this to prevent the formation of α-TCP and to ensure the completeness of the solid-state reaction [49]. The powder X-ray diffraction (PXRD) patterns were performed using Rigaku SmartLab SE: 3 kW sealed X-ray tube, D/teX Ultra 250 silicon strip detector, vertical type θ-θ geometry, and HyPix-400 (2D HPAD) detector (Rigaku, Tokyo, Japan). PXRD data were collected at room temperature (RT) in the 2θ range between 3° and 80° with a step interval of 0.02°. The LeBail decomposition was applied to obtain unit cell parameters and phase composition. Refinements of the structures were made by the Rietveld method using the JANA2006 software (Praha, Czech Republic) [50].

#### 2.2.2. Fourier-Transform Infrared (FT-IR) Study

IR spectra were recorded on a Nicolet 6700 FT-IR spectrometer (Thermo Fisher Scientific Inc., Hillsboro, OR, USA, 2010) in the 4000–400 cm^−1^ region (KBr tablets) with a spectral resolution of 1 cm^−1^.

#### 2.2.3. Second Harmonic Generation Study

The second harmonic generation (SHG) signal was measured with a Q-switched YAG:Nd laser (home-made, Moscow, Russia) at λ_ω_ = 1064 nm in the reflection mode.

#### 2.2.4. Differential Scanning Calorimetry Measurements

Differential scanning calorimetry (DSC) measurements were performed on a thermal analyzer SDT Q600 V8.1 Build 99 (TA Instruments, New Castle, DE, USA) with a Pt/Rh thermocouple in the temperature range from 25 to 1400 °C. The heating rate was 5°/min.

#### 2.2.5. The Release Behavior of Ions from β-TCP

The soaking of the ceramic samples in Ringer solution was applied to investigate the in vitro release of Co^2+^ and Ni^2+^ ions. In detail, 0.33Ni-TCP, 1.00Ni-TCP, 0.33Co-TCP, and 1.00Co-TCP samples were pressed into pallets (*d* = 0.5 cm, *m*~0.5 g) and calcinated at 950 °C to obtain the ceramics. The ceramics were soaked in the Ringer solution in a shaking bath at a rate of 60 rpm at 37 °C for 28 days. The sample’s extracts were taken into solution after 1, 3, 7, 14, and 28 days of soaking. The accumulative release amount of Co^2+^ or Ni^2+^ ions was measured using inductively coupled plasma optical emission spectroscopy (ICP-OES, 720-ES axial spectrometer, Agilent Technologies, NY, USA). The obtained data were reported as mean ± standard deviation.

#### 2.2.6. Isolation and Growth of aMSCs

Adipose tissue-derived mesenchymal stem cells were isolated from the adipose tissue of 3-month-old female lambs taken at the slaughterhouse. Adipose tissue was sterilely removed from the animal and placed in sterile PBS (Phosphate Buffed Saline, Gibco, UK), supplemented with antibiotics (100 IU Penicillin, 100 μg/mL Streptomycin, and 0.25 μg/mL Amphotericin B—all purchased from Gibco, UK), and quickly transported to the laboratory. The tissue was minced into small fragments and subsequently digested with Collagenase IA (Sigma-Aldrich, Gillingham, UK) at 0.1% in PBS (Phosphate Buffed Saline, Gibco, UK) at 37 °C for 60 min with slow stirring. At the end of the digestion, DMEM (Gibco, UK) was added with 10% FCS (Fetal calf serum, Gibco, UK) and subsequently centrifuged at 1200 g for 10 min. The pellet was washed 3 times in PBS, re-suspended in DMEM with 10% FCS, seeded in 75 cm^2^ flasks, and incubated at 37 °C with 5% CO_2_. DMEM with 10% FCS was replaced every 2 days, and cells were observed daily with an inverted light microscope (Nikon eclipse TE2000-U).

#### 2.2.7. MTT Assay

The activity of Ni and Co was evaluated by studying the viability of aMSC at the second passage for 24 h using an MTT assay. The Ca_10.5−*x*_Ni*_x_*(PO_4_)_7_ (with *x* = 0, 0.33, 0.67, 1.0, 1.33, and 1.58) and Ca_10.5−*x*_Co*_x_*(PO_4_)_7_ (with *x* = 0, 0.33, 0.5, 0.67, and 1.0) powders were autoclaved at 121 °C for 20 min. The aMSCs were distributed in 96-well plates at a concentration of 20,000 cells/mL in DMEM with 10% FCS and incubated at 37 °C in 5% CO_2_. After 24 h of incubation, the culture medium was replaced with DMEM 10% FCS containing 0.1 mg/mL of TCP, Ni (0.33, 0.67, 1.0, 1.33, and 1.58), and Co (0.33, 0.5, 0.67, and 1.0) in TCP and incubated at 37 °C with 5% CO_2_. Each experimental condition was repeated in triplicate. After 24 h of incubation, the DMEM was removed and 0.3 mL/well of a solution of MTT (3-[4,5-dimethylthiazol-2-yl]-2,5-diphenyl-tetrazolium bromide, Sigma-Aldrich, UK) 0.5 mg/mL was added. After 3 h of incubation at 37 °C with 5% CO_2_, the MTT solution was replaced with 1.5 mL of ethanol (Sigma-Aldrich, UK). The solubilized formazan was measured by a biophotometer (Eppendorf, Hamburg, Germany) at 600 nm. Cell viability was proportional to the intracellular reduction of tetrazolium salts by the mitochondrial enzyme succinate dehydrogenase, which formed blue formazan crystals.

#### 2.2.8. Differentiation in the Osteogenic Lineage

The differentiating potential of the aMSCs at the second passage was evaluated in the presence of 0.1 mg/mL pure TCP, Ni-TCP (*x* = 0.33, 0.67, 1, 1.33, and 1.58), and Co-TCP (*x* = 0.33, 0.5, 0.67, and 1) powders. The aMSCs were distributed in 6-well plates at a concentration of 20 000 cells/mL in DMEM with 10% FCS and incubated at 37 °C in 5% CO_2_. After 24 h, the medium was removed from each well, and the medium for osteogenic differentiation was added (DMEM with FCS 10%, ascorbic acid 50 µg/mL, β-glycerophosphate 10 mM, and dexamethasone 10^−7^ M) with the different substrates. Three wells were treated for each experimental condition. The aMSCs subjected to differentiation, but without the addition of substrate, represented the positive control. The aMSC grown only in DMEM and 10% FCS represented the negative control. After 3 weeks of osteogenic differentiation, calcium deposits were detected by staining with Alizarin Red S (Carlo Erba, Cornaredo, Italy). The aMSCs were fixed for 1 h at room temperature in 70% ethanol, washed with distilled water, and stained with 2% Alizarin Red S for 30 min. After 4 washes with distilled water, the calcium deposits turn orange-red.

#### 2.2.9. Antimicrobial Activity

The study of the antimicrobial activity of Ni-TCP (*x* = 0.33, 0.67, 1, 1.33, and 1.58) and Co-TCP (*x* = 0.33, 0.5, 0.67, and 1) powders was performed using *E. coli*, *S. aureus*, *E. faecalis*, *P. aeruginosa* bacteria strains, and *C. albicans* fungus. Prior to testing, all substrates were autoclaved at 121 °C for 20 min. All substrates were added in the Brain Heart Infusion (BHI, DIFCO, Sparks, MD, USA) at a concentration of 0.1 mg/mL. The positive controls were performed by culturing the microorganisms in the BHI and TCP at 0.1 mg/mL. The bacteria (*E. coli*, *S. aureus*, *E. faecalis,* and *P. aeruginosa*) were grown at 37 °C, while *C. albicans* was grown at 28 °C with 24 h of stirring. Each trial was repeated in triplicate. The growth of microorganisms was evaluated by reading the optical density at 600 nm (OD600) with a D30 biophotometer (Eppendorf, Hamburg, Germany).

#### 2.2.10. Statistical Analysis

All the experiments were performed in triplicate. The MTT assay on the grown aMSC and the growth rates of *C. albicans*, *E. coli*, *E. faecalis*, *S. aureus,* and *P. aeruginosa* were expressed as mean ± standard deviation (S.D.) and analyzed by the nonparametric Dunnett test for multiple comparisons (the software Sas Jmp Statistical Discovery v14 pro, Milan, Italy). *p*-values ≤ 0.05, ≤0.01, and ≤0.001 were considered statistically significant, as indicated in the figure legends.

## 3. Results

### 3.1. PXRD Study

**Figure 1:**
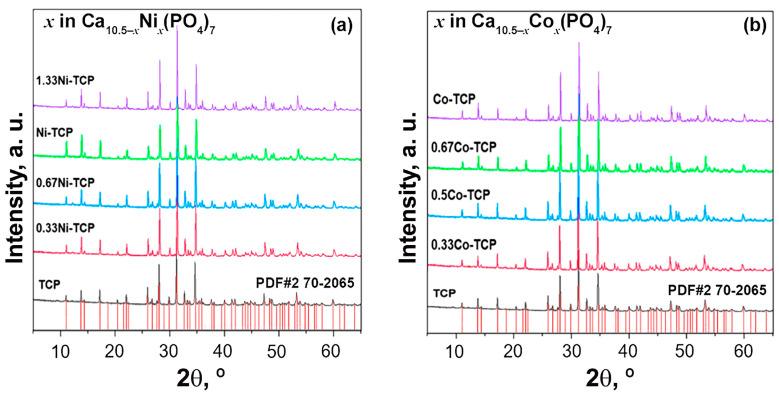
PXRD patterns of the synthesized Ca_10.5−*x*_Ni*_x_*(PO_4_)_7_ with 0 ≤ *x* ≤ 1.33 (**a**) and Ca_10.5−*x*_Co*_x_*(PO_4_)_7_ with 0 ≤ *x* ≤ 1 (**b**) along with the reference card form PDF-2 database No. 70-2065 Ca_3_(PO_4_)_2_.

The PXRD patterns of Ca_10.5−*x*_Ni*_x_*(PO_4_)_7_ and Ca_10.5−*x*_Co*_x_*(PO_4_)_7_ phosphates synthesized in this work are presented in Figure 1. The data analysis revealed that all the powder samples belong to the β-TPC structural type. No impurities of the apatite-type or pyrophosphate phases were detected, confirming the complete reaction. Up to *x* = 1.0, no other reflections from possible impurities were detected in the Ni-TCP and Co-TCP series (Figure 1).

**Figure 2:**
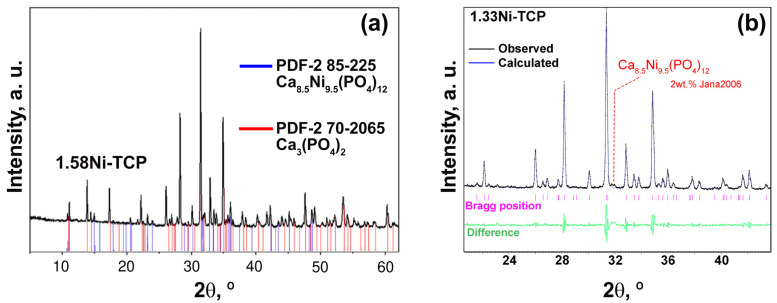
PXRD patterns of 1.58Ni-TCP (**a**) and 1.33Ni-TCP (**b**) samples. The impurity phase Ca_8.5_Ni_9.5_(PO_4_)_12_ (PDF#2 card No. 85-225) is highlighted.

In the 1.58Ni-TCP sample, the presence of the Ca_8.5_Ni_9.5_(PO_4_)_12_ impurity phase with the monoclinic space group *C*2/*c* and the unit cell parameters *a* = 22.8190 Å, *b* = 9.9440 Å, and *c* = 16.9820 Å [36] was observed (Figure 2a). Quantitative analysis using Jana2006 [50] revealed the presence of 7 wt.% of the impurity in the 1.58Ni-TCP sample.

In [46], it was shown that the 1.35Ni-TCP sample contains an impurity of the hydroxyapatite (HAP) phase. It should be noted that the HAP phase may form only in soft conditions of chemistry methods of synthesis, such as precipitation [43], sol–gel [46], or hydrothermal. The careful XRD study of the 1.33Ni-TCP sample also showed the presence of an impurity phase (Figure 2b), with a calculated content not exceeding 2 wt.%. Hence, the limit composition of the Ca_10.5−*x*_Ni*_x_*(PO_4_)_7_ solid solution in this study was determined as Ca_9.5_Ni_1.0_(PO_4_)_7_.

Similar results were observed in the Ca_10.5−*x*_Co*_x_*(PO_4_)_7_ solid solution, where even a slight excess of Co^2+^ doping β-TCP more than 9.5 mol.% leads to the formation of β-Ca_2_P_2_O_7_ impurity, as reported in [44,51]. Therefore, the solubility limit of Co^2+^ and Ni^2+^ ions in the Ca_10.5−*x*_Co*_x_*(PO_4_)_7_ solid solution with the β-TCP structure corresponds to *x* = 1.00.

It is worth noting that in the isostructural Ni-doped vanadates Ca_10.5−*x*_Ni*_x_*(VO_4_)_7_ with the β-TCP-type structure, the limit of the solid solution was found at *x* = 0.72(2) [52]. This difference can be attributed to the enlarged unit cell size in the vanadates, which imposes restrictions on the isomorphic substitution of Ca^2+^ by noticeably smaller Ni^2+^ ions.

**Figure 3:**
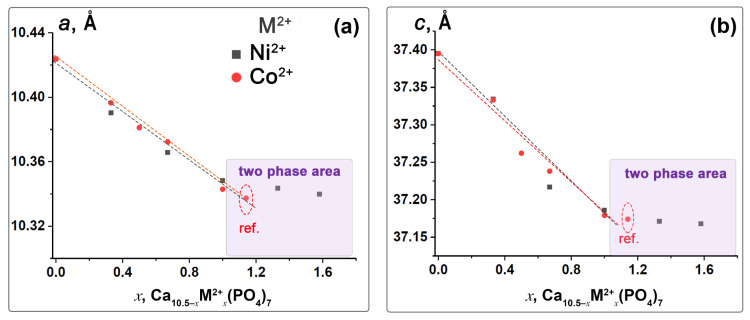
Dependence of the unit cell parameters *a* (**a**) and *c* (**b**) in Ca_10.5−*x*_*M_x_*(PO_4_)_7_, *M* = Ni^2+^ (gray) and Co^2+^ (red). The ref. means the data on the unit cell parameters from ref. [44].

The monotonic decrease in the unit cell parameters and volume in Ni-TCP and Co-TCP solid solutions can be attributed to the smaller ionic radii of Ni^2+^ (*r*_VI_ = 0.69 Å) and Co^2+^ (*r*_VI_ = 0.74 Å) doping ions compared to the host Ca^2+^ ions (*r*_VI_ = 1.00 Å). This decrease is also influenced by an increase in the concentrations of Ni^2+^ and Co^2+^ ions (Table 2 and Figure 3). The deviation of the unit cell parameters from the linear dependence and Vegard’s law [53] at *x* > 1.00 (Figure 3) indicates the saturation of the structure by Ni^2+^ ions.

It is worth noting that no reflections from the α-TCP phase were detected, suggesting that Ni- and Co-doping stabilized the β-TCP phase. This stabilization effect of Co^2+^ ions was previously observed in Ca_10_Li(PO_4_)_7_: Co^2+^-based scaffolds, where up to 10 mol.% of Co^2+^ prevented the formation of apatite-phase even after soaking in a simulated body fluid (SBF) for 30 days [37]. This effect is attributed to the structural differences between the α- and β-TCP phases. α-TCP is characterized by larger Ca-polyhedra compared to β-TCP [54], while Ni^2+^ and Co^2+^ ions prefer octahedral coordination.

### 3.2. Crystal Structure Refinement

The PXRD patterns were used to refine the crystal structure. Previously, it was shown that phosphates with the β-TCP structure can have either a polar or non-polar structure with a variety of space groups [55]. Based on the crystal’s chemical structure, the observed space groups are:

- *R*3*c* for Ca_3_(PO_4_)_2_:*M*^+^, Ca_3_(PO_4_)_2_:*M*^2+^, Ca_3_(PO_4_)_2_:*M*^3+^ (Ca_9_□*M*^3+^(PO_4_)_7_) [56]
- $R\bar{3}c$ for Ca_3_(PO_4_)_2_:*M*^2+^*M*^3+^
- $R\bar{3}m$ for Sr_3_(PO_4_)_2_:*M*^2+^*M*^3+^
- *C*2/*m* Sr_9_□*R*^3+^(PO_4_)_7_ (*R*^3+^ = Sc, Cr, Fe, Ga)
- *C*2/*c* Sr_9_□In(PO_4_)_7,_

where *M*^+^ = Li, Na, K, and Ag; *M*^2+^ = Mg, Zn, and Fe^2+^; and □—is a vacancy.

It should be noted that Sr_3_(PO_4_)_2_:*M*^+^*M*^3+^ phosphate exhibits a palmierite-type structure, while Sr_9_□REE^3+^(PO_4_)_7_ cannot be obtained as a single-phase compound. The parent phase has a $P{\bar{3}}_{1}m$ space group; however, no representatives of the mineral or synthetic compounds have been found at this time.

**Figure 4:**
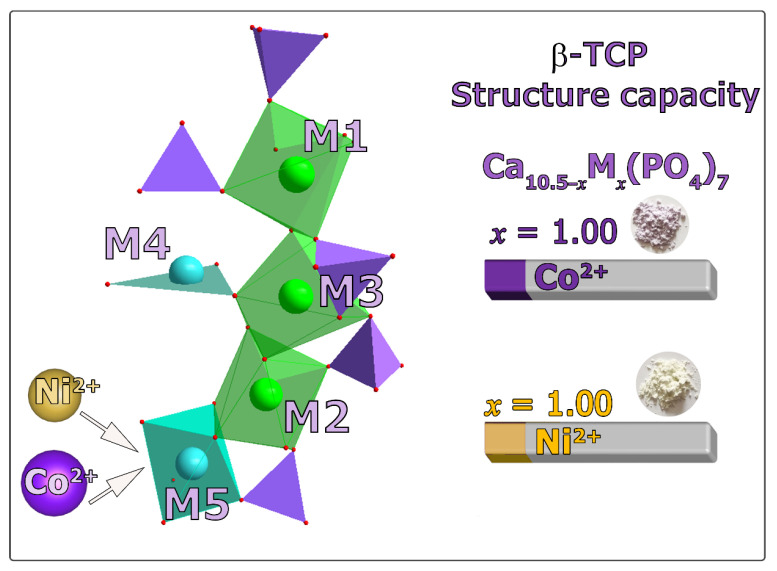
The schematic representation of the β-TPC structure and the structure saturation by Ni^2+^ or Co^2+^ ions. The insets show the images of the Ni-TCP and Co-TCP powder samples.

According to the SHG study, the Ni-TCP and Co-TCP samples have a non-polar structure. The SHG signal value gradually decreases from 2.5 units, which is related to the SiO_2_ etalon in pure β-Ca_3_(PO_4_)_2_ [10], to 0.8 units in Co-TCP and 1.0 in 1.33Ni-TCP due to the presence of the powder’s color (Figure 4, inset images). The same decrease in the SHG signal was observed in Cu^2+^-doped β-TCP [10] and was also related to the color. Nevertheless, the space group chosen for the refinement was *R*3*c* (No. 161). Therefore, the starting model for the refinement was based on β-Ca_3_(PO_4_)_2_ [57]. A pseudo-Voigt function was used to fit the reflection profiles. The main crystallographic data and experimental details of Ca_10.5−*x*_Ni*_x_*(PO_4_)_7_ and Ca_10.5−*x*_Co*_x_*(PO_4_) are listed in Table S1 of the Supporting Information. According to the refinement, Co^2+^ and Ni^2+^ sequentially occupy the M5 site as their concentrations increase. Supporting Information contain atomic coordinates, displacement parameters, and site-occupancy factors in Tables S2–S10. The final results of the refinement are presented in Figures S1–S9 of the Supporting Information.

### 3.3. FT-IR Study

**Figure 5:**
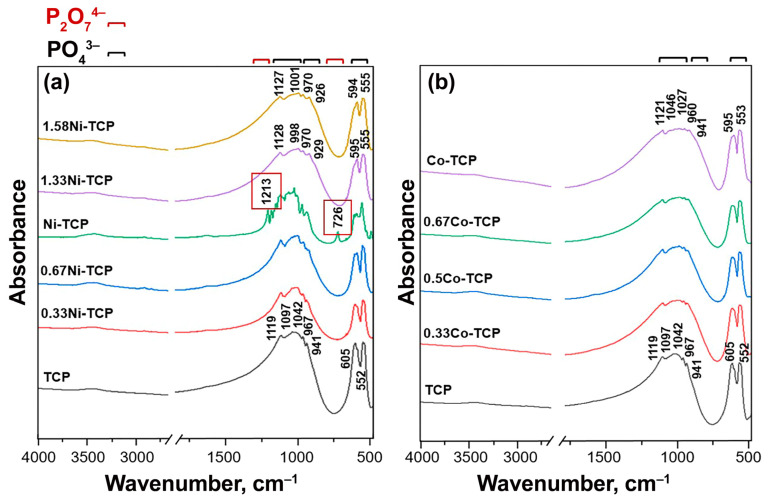
FTIR spectra of TCP, Ni-TCP (*x* = 0.33, 0.67, 1, 1.33, and 1.58) (**a**), and Co-TCP (*x* = 0.33, 0.5, 0.67, and 1) (**b**).

The Ca_10.5−*x*_Ni*_x_*(PO_4_)_7_ and Ca_10.5−*x*_Co*_x_*(PO_4_)_7_ series were investigated using FT-IR spectroscopy to determine functional groups (Figure 5). It was found that most phosphates only contain the phosphate group PO_4_^3−^ [58,59]. The spectra of the Co-series samples were almost identical. Only the 1.0Ni-TCP sample in the Ni-series showed slight differences, with bands at 1213 cm^−1^ and 726 cm^−1^ assigned to P_2_O_7_^4−^ [28]. These differences were attributed to trace amounts of impurities in the initial components, as the intensity was low. The results of the PXRD phase analysis confirmed the presence of an extremely small amount of pyrophosphate impurity (pyrophosphates of calcium or nickel/cobalt were not detected).

No significant differences were observed between the spectra of the nickel and cobalt series. The characteristic bands from inorganic carbonate ions (1465–1415 and 870 cm^−1^) or H_2_O molecules or OH^−^ groups (4000–3000 cm^−1^) are absent in the IR spectra of the investigated samples [60,61]. A comparative analysis of the obtained spectra and those reported in the literature confirms that the composition of the obtained substances includes β-TCP [62].

### 3.4. Phase Transitions

**Figure 6:**
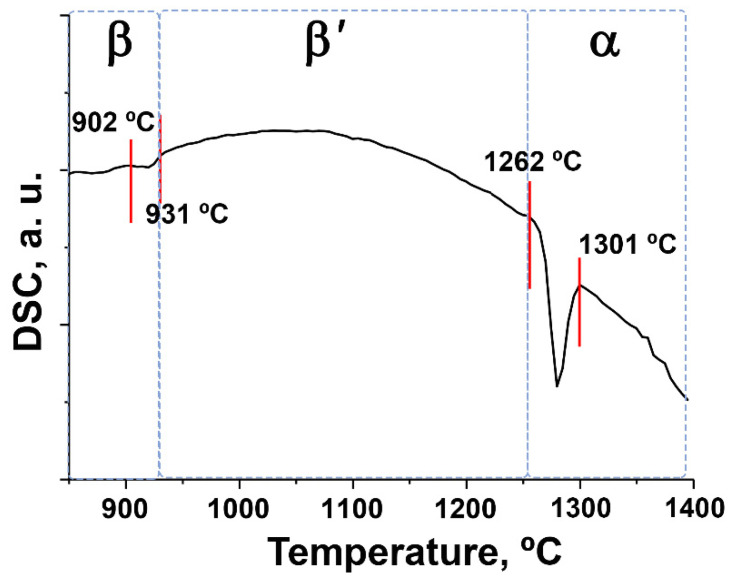
DSC curve for the 0.67Ni-TCP sample in the heating mode.

**Figure 7:**
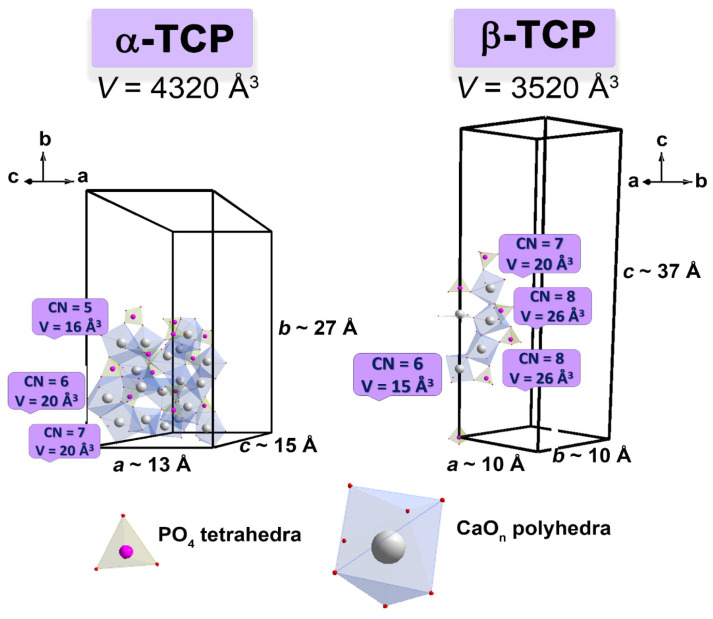
The projections of the α-TCP and β-TCP crystal structures.

Figure 6 shows the DSC measurements for the 0.67Ni-TCP sample. According to the SGH study, all synthesized compounds have polar crystal structures, and the Ni-TCP and Co-TCP series exhibit ferroelectric properties. Previous studies have attempted to delay the β→α phase transition. Projections of α-TCP and β-TCP, as well as their comparison, are shown in Figure 7. However, two sequential phase transitions occur: the β → β′ and the further β′ → α transition (Figure 6).

The first transition, β → β′, corresponds to the transformation from a ferroelectric to a paraelectric phase. This transformation is accompanied by the appearance of the center of symmetry and the re-orientation of tetrahedral PO_4_ fragments along the *c*-axis. The thermal effect of this transition is relatively small. The second transformation, β′ → α, results in significant changes in the structures, including changes in the number of crystal sites and their coordination. Therefore, the thermal effect of β′ → α is noticeably higher (Figure 6).

It is worth noting that the incorporation of small ions, such as Mg^2+^ or Zn^2+^, in the octahedral M5 site delays the temperature of this transition compared to pure β-TCP. This delay exhibits concentration-dependent behavior. The transition to the α-phase is accompanied by an increase in cell volume, leading to volumetric expansion (up to 7%, Figure 7). This expansion reduces shrinkage and prevents further densification of β-TCP-based ceramics [63]. The temperature of the thermal treatment plays a crucial role in obtaining bulk ceramics [63].

For the 0.67Ni-TCP sample, the transition to the α-phase occurs at 1262 °C, while for β-TCP, it was found at 1196 °C [64]. A similar tendency was shown for the Co^2+^-doped β-TCP ceramics, and in 2 mol.% Co^2+^-doped β-TCP (Ca_10.29_Co_0.21_(PO_4_)_7_ or Ca_2.94_Co_0.09_(PO_4_)_2_), the density of the ceramics was significantly improved compared to pure β-TCP [38].

### 3.5. Ion Release Behavior and Soaking of the Ceramics

**Figure 8:**
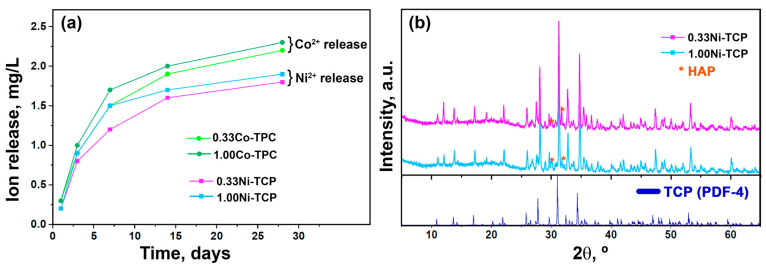
(**a**) Co^2+^ and Ni^2+^ accumulative release profiles from Ca_10.5−*x*_M*_x_*(PO_4_)_7_ ceramics, *x* = 0.33 and 1.00, and (**b**) XRD patterns of 0.33Ni-TCP and 1.00Ni-TCP after 28 days of soaking and a PDF-4 card of β-TCP.

The results of the ceramic’s soaking in the Ringer solution for 28 days are shown in Figure 8a. The different concentrations of doping ions do not significantly affect the ion release profile of the samples. It should be noted that the release concentration of Co^2+^ ions is higher than that of Ni^2+^ ions (Figure 8a). This fact can be related to the larger ionic radii of Co^2+^ rather than Ni^2+^ ions and their lower binding in the units of the crystal lattice.

The XRD patterns on the surface of the ceramics after 28 days of soaking in the Ringer solution are shown in Figure 8b. The HAP phase was formed according to the reaction (3):

10Ca_10.5−*x*_Ni*_x_*(PO_4_)_7_ + 6H_2_O → 3Ca_10−*x*_Ni*_x_*(PO_4_)_6_(OH)_2_ + 2H_3_PO_4_ (3)

The intensity of the reflexes belonging to the HAP phase is higher in the 0.33Ni-TCP sample than in the 1.00-Ni-TCP. Therefore, it can be concluded that a higher concentration of Ni^2+^ ions in the M5 site leads to stabilization of the β-TCP structure and prevents rapid HAP formation.

### 3.6. Bioactive Properties

#### 3.6.1. Cell Viability Determination by MTT Assay

**Figure 9:**
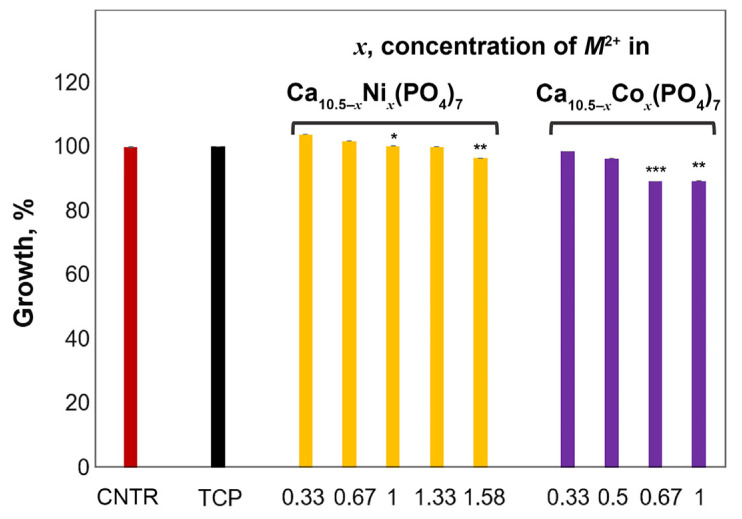
MTT assay on aMSC grown for 24 h in the presence of 0.1 mg/mL pure TCP, Ni-TCP (0.33, 0.67, 1.0, 1.33, and 1.58), and Co-TCP (0.33, 0.5, 0.67, and 1.0). The reported values of % cell growth were obtained from three independent experiments and expressed as mean percentage values ± S.D. CTRL (cell control) values correspond to 100%. *p*-values (Dunnett test): *p* < 0.05 *, *p* < 0.01 **, *p* < 0.001 *** versus CTRL.

The viability of the aMSC grown for 24 h in the presence of 0.1 mg/mL TCP, Ni (0.33, 0.67, 1.0, 1.33, and 1.58), and Co (0.33, 0.5, 0.67, and 1.0) in β-TCP was evaluated by the MTT assay. Table 3 shows the mean values of three experiments of the aMSC growth percentage and the standard deviation (SD), related to the optical density (OD) at 600 nm. The values correspond to the growth of aMSC with respect to the 100% cell growth of the control. The results of the MTT test are reported in Table 3 and in Figure 9.

As reported in Figure 9, pure β-TCP and doped TCP phosphates do not have an inhibiting effect on cell growth. The addition of Ni-TCP (0.33, 0.66, 1.0, and 1.33) has no toxic effects on cell growth; in fact, aMSCs grew to 103.95% in the presence of 0.33Ni-TCP, to 101.90% in the presence of 0.67Ni-TCP, to 100.38% in the presence of 1.0Ni-TCP, and to 100.11% in the presence of 1.33Ni-TCP. Only in the presence of 1.58Ni-TCP does the growth of aMSC drop to 96.53%. This effect is likely related to the presence of the impurity Ca_8.5_Ni_9.5_(PO_4_)_7_ phosphate.

The aMSCs grown in the presence of Cc-doped β-TCP phosphates are characterized by lower values. Thus, the aMSCs in the presence of 0.33Co-TCP grew to 98.68% of the control, in the presence of 0.5Co-TCP to 96.47%, in the presence of 0.67Co-TCP to 89.38%, and in the presence of 1.0Co-TCP to 89.43%. To summarize, according to the MTT test, in all the experimental conditions, the inhibition of growth of aMSCs was statistically not significant with respect to the control.

#### 3.6.2. Differentiation in the Osteogenic Lineage

**Figure 10:**
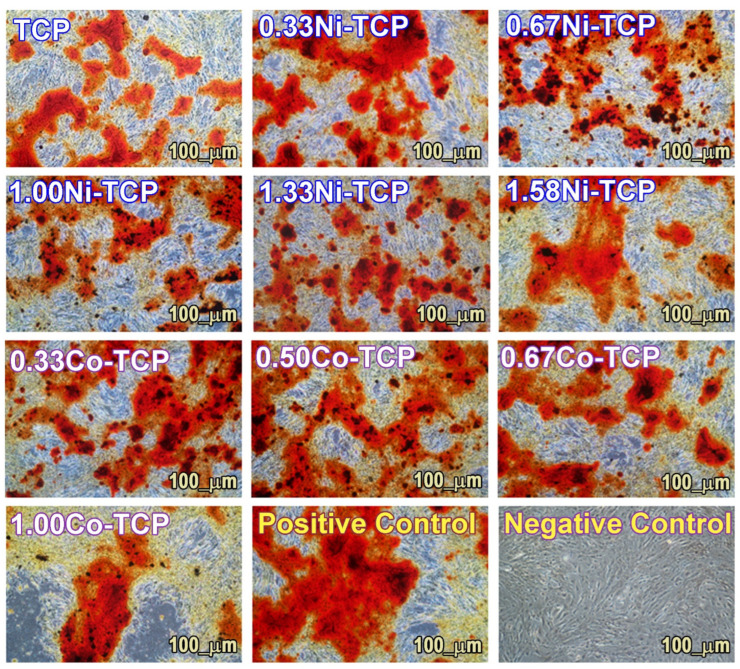
aMSC differentiated into the osteogenic lineage in the presence of TCP, Ni-TCP, and Co-TCP samples. Positive control are aMSC differentiated in the absence of substances, and negative control are non-differentiated aMSC. All tests were stained with Alizarin Red S. The images were taken by means of an inverted microscope at ×10 magnification.

The differentiation potentials in the osteogenic aMSC lineage were evaluated in the presence of 0.1 mg/mL of TCP, Ni (0.33, 0.67, 1.0, 1.33, and 1.58), and Co (0.33, 0.5, 0.67, and 1.0) in the β-TCP (Figure 10). Red staining indicates differentiation since Alizarin Red stains calcium deposits as red. Positive control is related to aMSC differentiated in the absence of substances (Figure 10), while negative control is related to non-differentiated aMSC grown in DMEM with 10% FCS (Figure 10). As shown in Figure 10, differentiation of aMSCs in the osteogenic lineage took place in all the experimental conditions, and all the samples are comparable to the positive control (Figure 10).

This qualitative test confirms the non-toxicity of the prepared samples at different concentrations of Ni and Co and their ability to non-interfere with the osteogenic potential of aMSCs. In fact, aMSCs grew and differentiated well in all the experimental conditions.

### 3.7. Inhibition of the Growth of Microorganisms

**Figure 11:**
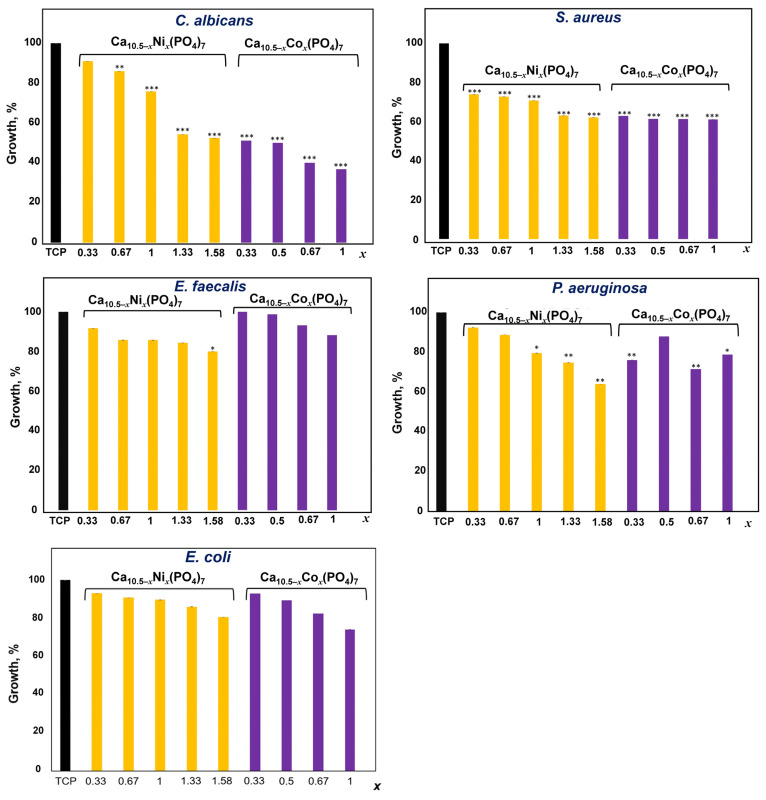
Growth rate and standard deviation of *C. albicans*, *E. coli*, *E. faecalis*, *S. aureus,* and *P. aeruginosa* grown in the presence of Ni (0.33, 0.67, 1.0, 1.33, and 1.58) and Co (0.33, 0.5, 0.67, and 1.0)-doped TCP. TCP represents the positive control. The reported values were obtained from three independent experiments and expressed as mean percentage values ± S.D., in comparison to TCP values corresponding to 100%. *p*-values (Dunnett test): *p* < 0.05 *, *p* < 0.01 **, *p* < 0.001 *** versus TCP.

The results of the growth of microorganisms (*E. coli*, *S. aureus*, *E. faecalis*, *P. aeruginosa,* and *C. albicans*) in the presence of different concentrations of Ni (0.33, 0.67, 1.0, 1.33, and 1.58) and Co (0.33, 0.5, 0.67, and 1.0) in TCP are reported in Figure 11 in graphs related to the single microorganism. The positive control is represented by each single microorganism grown in the presence of TCP. The growth of each organism was evaluated after 24 h of incubation at their respective optimal growth temperatures. The growth rate and standard deviation (ST) are obtained from the mean of three independent experiments.

As shown in Figure 11, the growth of the *C. albicans* fungus is significantly and progressively inhibited by the presence of Ni, passing from an inhibition of about 9% for 0.33Ni-TCP to about 48% for 1.58Ni-TCP compared to the control (100%). In particular, in the presence of 0.33Ni-TCP, the *C. albicans* fungus grows by 91.07%, in the presence of 0.67Ni-TCP—by 86.12%, in the presence of 1.00Ni-TCP—by 75.84%, in the presence of 1.33Ni-TCP—by 54.36%, and in the presence of 1.58Ni-TCP—by 52.48%, with respect to the control. More significant is the inhibition of growth of *C. albicans* in the presence of Co ion substitution, which goes from about 49% in the presence of 0.33Co-TCP to an inhibition of 63% in the presence of 1.0Co-TCP. In detail, in the presence of 0.33Co-TCP, *C. albicans* grows by 51.27%, in the presence of 0.5Co-TCP—by 50.16%, in the presence of 0.67Co-TCP—by 40%, and in the presence of 1.0Co-TCP—by 36.91%, compared to the growth of the control.

In the presence of Ni, the growth of the *E. coli* bacteria strain is inhibited less significantly than that of the *C. albicans* fungus, passing from an inhibition of about 7% for 0.33Ni-TCP to about 20% for 1.58Ni-TCP compared to the control. In detail, *E. coli* in the presence of 0.33Ni-TCP grows by 93.07%, in the presence of 0.67Ni-TCP—by 90.94%, in the presence of 1.0Ni-TCP—by 89.67%, in the presence of 1.33Ni-TCP—by 86.09%, and in the presence of 1.58Ni-TCP—by 80.59%, with respect to the control. In the presence of Co, the inhibition of *E. coli* growth goes from about 7% for 0.33Co-TCP to an inhibition of 26% for 1.0Co-TCP. In detail, in the presence of 0.33Co-TCP, *E. coli* grows by 92.83%, in the presence of 0.5Co-TCP—by 89.33%, in the presence of 0.67Co-TCP—by 82.43%, and in the presence of 1.0Co-TCP—by 74.19%, compared to the growth of the control (Figure 11).

In the presence of Ni, the growth of *E. faecalis* is inhibited, passing from an inhibition of about 8% for 0.33Ni-TCP to about 20% for 1.58Ni-TCP, with respect to the control. In detail, in the presence of 0.33Ni-TCP, *E. faecalis* grows by 91.65%, in the presence of 0.67Ni-TCP—by 85.68%, in the presence of 1.0Ni-TCP—by 85.68%, in the presence of 1.33Ni-TCP—by 84.35%, and in the presence of 1.58Ni-TCP—by 79.88%, related to the control. In the presence of Co, the growth inhibition of *E. faecalis* goes from about 0% for 0.33Co-TCP to an inhibition of 12% for 1.0Co-TCP. In detail, in the presence of 0.33Co-TCP, the growth of *E. faecalis* is not inhibited; in the presence of 0.5Co-TCP, it grows by 98.67%; in the presence of 0.67Co-TCP, it grows by 93.16%; and in the presence of 1.0Co-TCP, it grows by 88.17%, compared to the control (Figure 11).

In the presence of Ni, the growth of *S. aureus* is inhibited, passing from an inhibition of about 26% for 0.33Ni-TCP to about 38% for 1.58Ni-TCP with respect to the control. In detail, in the presence of 0.33Ni-TCP, *S. aureus* grows by 73.88%, in the presence of 0.67Ni-TCP—by 72.71%, in the presence of 1.0Ni-TCP—by 70.76%, in the presence of 1.33Ni-TCP—by 62.99%, and in the presence of 1.58Ni-TCP—by 62.07%, related to the control. In the presence of Co, the growth inhibition of *S. aureus* goes from about 37% for 0.33Co-TCP to an inhibition of 39% for 1.0Co-TCP. In detail, in the presence of 0.33Co-TCP, the growth of *S. aureus* increased by 62.77%; in the presence of 0.5Co-TCP, it grew by 61.30%, in the presence of 0.67Co-TCP, it grew by 61.22%, and in the presence of 1.0Co-TCP, it grew by 61.11%, compared to the growth of the control (Figure 11).

In the presence of Ni, the growth of *P. aeruginosa* is inhibited, passing from an inhibition of about 8% for 0.33Ni-TCP to about 36% for 1.58Ni-TCP with respect to the control. In detail, in the presence of 0.33Ni-TCP, *P. aeruginosa* grows by 92.40%, in the presence of 0.67Ni-TCP—by 88.82%, in the presence of 1.0Ni-TCP—by 79.60%, in the presence of 1.33Ni-TCP—by 74.81%, and in the presence of 1.58Ni-TCP—by 64.18%, related to the control. In the presence of Co, the inhibition of growth of *P. aeruginosa* goes from about 24% for 0.33Co-TCP to an inhibition of 31% for 1.0Co-TCP. In detail, in the presence of 0.33Co-TCP, the growth of *P. aeruginosa* is 76.06%; in the presence of 0.5Co-TCP, it grows by 87.95%; in the presence of 0.67Co-TCP, it grows by 71.61%; and in the presence of 1.0Co-TCP, it grows by 78.71, compared to the growth of the control (Figure 11).

Therefore, as shown in Figure 11, the inhibition of growth of *C. albicans* is significant when exposed to 0.66Ni-TCP, 1Ni-TCP, 1.33Ni-TCP, 1.58Ni-TCP, 0.33Co-TCP, 0.5Co-TCP, 0.67Co-TCP, and 1Co-TCP, compared to the control sample. For *E. coli*, the inhibition of growth is significant in the presence of 1.58Ni-TCP, 0.67Co-TCP, and 1Co-TCP, compared to the control. The inhibition of growth of *E. faecalis* is significant when exposed to 1.58Ni-TCP, compared to the control. The growth of *S. aureus* is significantly inhibited in all the experimental conditions. The inhibition of growth of *P. aeruginosa* is significant when exposed to 1Ni-TCP, 1.33Ni-TCP, 1.58Ni-TCP, 0.33Co-TCP, 0.67Co-TCP, and 1Co-TCP, compared to the control.

It was found that the cytotoxicity of substituted TCPs depends both on their concentration and on the content of dopants; at various nickel (0.33, 0.67, 1.0, 1.33, and 1.58) and cobalt (0.33, 0.5, 0.67, and 1.0) concentrations, substituted TCPs do not exhibit cytotoxicity at their concentration of 0.1 mg/mL.

## 4. Discussion

Ni- and Co-doped series of phosphates with the β-TCP structure were synthesized using the solid-state method, and ceramics were obtained. This study demonstrated that the saturation of the β-TCP structure occurs at 9.5 mol/% for Co^2+^ ions and Ni^2+^ ions, corresponding to the chemical formula Ca_9.5_*M*^2+^(PO_4_)_7_, where *M*^2+^ = Ni^2+^ and Co^2+^. It should be noted that in the isostructural copper-doped Ca_10.5−*x*_Cu*_x_*(PO_4_)_7_ solid solution, the saturation of the β-TCP structure by Cu^2+^ ions was found at 14.2 mol.% [10]. This difference can be attributed to the various crystal chemical environments of copper ions, while nickel and cobalt mainly occupy the octahedral coordination in inorganic salts. The Rietveld refinement performed in the present research revealed the preferential occupation of Ni^2+^ and Co^2+^ ions in the octahedral M5 sites due to their smaller ionic radii compared to the host Ca^2+^ ions. Consequently, after complete occupation of the M5 octahedra (at 9.5 mol/%), these ions are no longer part of the β-TCP structure and form the impurity phases. The obtained results are supported by previous studies [38,46], where impurities were also observed after M5 site saturation.

The incorporation of these ions postpones the phase transition to the α-TCP upon heating and to the HAP phase during the soaking of the ceramics. Therefore, doping β-TCP with Ni or Co stabilizes its structure. A similar effect has also been observed with other ions with small radii, such as Zn^2+^ or Mg^2+^ [65]. On the other hand, Sr^2+^ or Ba^2+^ ions induce the formation of the α-TCP phase.

This trend of increasing the structural stability of β-TCP also applies to the soaking of the ceramics. Increasing the concentration of Ni^2+^ or Co^2+^ postpones the dissolution–precipitation from β-TCP to the HAP phase. Prolonged preservation of the β-TCP structure promotes better osteoblast differentiation [66]. However, at a certain ratio of β-TCP and HAP (β-TCP/HAP 70:30), the two-phase compound shows favorable parameters for cell viability, such as mitochondrial activity, membrane integrity, and cell density, both in vitro and in vivo tests [67]. The results obtained in this work indicate that the release of Co^2+^ ions into solution is slightly easier than the release of Ni^2+^ ions during the soaking of the ceramics in the Ringer solution.

Based on the results of the MTT assay, it can be concluded that the CPs with Ni^2+^ and Co^2+^ substitutions are not toxic for aMSCs at a concentration of 0.1 mg/mL. Co^2+^ ions have a greater effect on the aMSC cell line and slightly suppress cell viability to 89.43% in the presence of 1.0Co-TCP. It should be noted that in the biphasic samples doped with Ni^2+^ (1.33Ni-TCP and 1.58Ni-TCP), the viability of aMSC cells did not drop below 96.53%. The difference in aMSC differentiation into the osteogenic lineage between the Co-doped and Ni-doped TCPs was not significant, so both metal ions promote the differentiation in a comparable way. The tested materials also do not inhibit cell differentiation into the osteogenic lineage and, consequently, are suitable for possible applications as bone substitutes.

Ni and Co have inhibitory properties for the growth of all the microorganisms investigated (four bacteria and one fungus). In particular, the inhibition effect is stronger against *C. albicans* in the presence of Ni and Co, *S. aureus* in the presence of Ni and Co, and *P. aeruginosa* in the presence of Ni. In all tests for inhibiting the growth of microorganisms, 1.58Ni-TCP showed the strongest results for four strains of bacteria. However, the 1.58Ni-TCP sample had an impurity phase, which may have contributed to this effect. The growth of microorganisms (*C. albicans*, *E. coli*, *S. aureus*, and *E. faecalis*) in the obtained Ni-TCP and Co-TCP showed a dependence on the dopant concentration in the TCP structure. However, the growth of *P. aeruginosa* in the series with cobalt has a chaotic order.

## 5. Conclusions

The Ca_10.5−*x*_Ni*_x_*(PO_4_)_7_ and Ca_10.5−*x*_Co*_x_*(PO_4_)_7_ solid solutions were synthesized through a high-temperature solid-phase reaction. It was found that the β-TCP structure reaches saturation at 9.5 mol/% of Co^2+^ and Ni^2+^ ions. The following series of samples were prepared and characterized: Ca_10.5−*x*_Ni*_x_*(PO_4_)_7_ (with *x* = 0, 0.33, 0.67, 1.0, 1.33, and 1.58) and Ca_10.5−*x*_Co*_x_*(PO_4_)_7_ (with *x* = 0, 0.33, 0.5, 0.67, and 1.0). The Rietveld method was used for the crystal structure refinement, showing the sequential occupation of the octahedral M5 sites by Ni^2+^ and Co^2+^ ions until they are fully complete. The incorporation of these ions into the β-TCP crystal structure delayed the phase transition to the α-phase during heating, resulting in structure stabilization.

The results from ceramics soaked in Ringer solution for 28 days indicate that the release concentration of Co^2+^ ions is slightly higher than that of Ni^2+^ ions released under the same conditions.

The prepared Ni- and Co-doped β-TCP series showed neither short-term cytotoxic effects on the aMSC cell line nor inhibition of the cell differentiation into the osteogenic lineage and, in the moderate term, promoted the osteogenic differentiation evidenced through the formation of mineralization nodules in the extracellular matrix. No significant difference was observed between the influence of Co and Ni on cells.

All the investigated Ni- and Co-doped β-TCP samples inhibited bacterial growth, depending on the concentration of the doping ion, and differently for various bacterial strains and fungi.

In the presence of Ni (for minimum and maximum limit concentrations, respectively): *C. albicans* growth was inhibited by 9–48%, *E. coli*—7–20%, *E. faecalis*—8–20%, *S. aureus*—26–38%, and *P. aeruginosa*—8–36%.

In the presence if Co (for minimum and maximum limit concentrations, respectively): *C. albicans* growth was inhibited by 49–63%, *E. coli*—7–26%, *E. faecalis*—0–12%, *S. aureus*—37–39%, and *P. aeruginosa* 24–31%.

The highest inhibitory activity was, therefore, about 40% against *S. aureus* bacteria and more than 60% against *C. albicans* fungus in both series. The combination of antibacterial activity and positive influence on the cells makes the prepared phosphates promising candidates for bone substitutes and anti-infection implants.

## Figures and Tables

**Table 1:** Literature data on physical and bioactive properties and optimum dopant concentration of Co^2+^- and Ni^2+^-doped CPs.

Formula	Doping Concentration Form	Physical Properties	Bioactive Properties	Optimum Concentration of Dopant	Ref.
Ca_9.5_Co(PO_4_)_7_ Ca_9+2*x*_Co_1+*x*_(PO_4_)_7_	*x* = 0.14 and 0.07 Powders	The impurity of the β-Ca_2_P_2_O_7_ phase in Ca_9+2*x*_Co_1+*x*_(PO_4_)_7_; Co^2+^ ions occupy the M5 site; The presence of Co^3+^ in the structure.	N/a	N/a	[44]
(Co_0.0*x*_Ca_1−0.0*x*_)_3_(PO_4_)_2_	*x* = 2 and 5 mol.% Ceramics	β-TCP is the main phase; Co^2+^ ions in the crystal structure suppressed the transition β-TCP → α-TCP.	Cytocompatibility to support HBMSC growth; Stimulation of VEGF expression in HBMSCs Enhance angiogenic properties	2 and 5 mol.%	[38]
(Co_0.0*x*_Ca_1−0.0*x*_)_3_(PO_4_)_2_	*x* = 1, 2, 5, and 10 mol.% Scaffolds	Biphasic β/α-TCP samples; Improved mechanical properties.	Boosted cell viability of bone marrow stromal cells; Promoted matrix mineralization and expression of osteogenic genes 0% Co^2+^ doping suppressed osteoblast differentiation	2 and 5 mol.%	[45]
Ca_10_Li(PO_4_)_7_	*x* = 0, 0.1, 0.25, 0.5, and 1 mol.% 3D-printed scaffolds	β-TCP main phase; Improved compressive strength (except 1 mol.%); Formation of HAP-phase after soaking for 28 days (except 1 mol.%).	Improved cell viability and rBMSCs cell proliferation Stimulation of tubule formation	0.25 mol.%	[37]
CPs:Co^2+^	0.1 and 20 mol.% Coatings deposited on poly (lactic acid) particles	N/a	stimulation of large blood vessel formation; Enhance vascularization in vivo.	N/a	[39]
β-Ca_3_(PO_4_)_2_:Ni	1.35 Powders	β-TCP with impurity of HAP phase; Reduction in the crystallite size.	1.35Ni-TCP displayed effective antimicrobial activity against *S. aureus* and *E. faecalis*; Improved bone regeneration	N/a	[46]
HAP:Ni^2+^	3 wt.% and 6 wt.%-doped HAP Biphasic calcium phosphate/graphene nanoplatelet (GNP) composites	HAP phase; Grain boundaries and reduction in crystallinity degree increase with increasing Ni^2+^ doping. Improvement in elastic modulus, microhardness, and fracture toughness.	6 wt.% Ni^2+^ showed cytotoxicity for hFOB cells	6 wt.% Ni^2+^ with 1.5 wt.% GNPs	[41]
Ca_2.9_Ni_0.1_(PO_4_)_2_	0.1 Powder	Ni^2+^ occupies the M5 site	N/a	N/a	[47]
β-Ca_3_(PO_4_)_2_:Ni	2.5, 5, 7.5, 10, and 12.5 mol.% Powders	Ni^2+^ occupies the M5 and M4 sites.	Hyperthermia effect; Significant toxicity at 10 and 12.5 mol.% of Ni^2+^.	Ni/Fe co-doping	[36]

**Table 2:** Chemical formula, *M*^2+^ concentration, unit cell parameters (*a*, *c*), and volume (*V*) in Ca_10.5−*x*_*M_x_*(PO_4_)_7_, *M* = Ni^2+^, Co^2+^.

Chemical Formula	*x*, Ni^2+^ or Co^2+^	*M*^2+^, mol.%	Sample Code	*a*, Å	*c*, Å	*V*, Å^3^
Ca_10.5_(PO_4_)_7_	0		TCP	10.4237(3)	37.395(2)	4063.1(7)
Ca_10.17_Ni_0.33_(PO_4_)_7_	0.33	3.1	0.33Ni-TCP	10.3903(5)	37.334(1)	4030.5(4)
Ca_9.83_Ni_0.67_(PO_4_)_7_	0.67	6.4	0.67Ni-TCP	10.3557(5)	37.217(8)	3991.2(3)
Ca_9.5_Ni(PO_4_)_7_	1.0	9.5	1.00Ni-TCP	10.3483(4)	37.186(9)	3982.1(8)
Ca_9.17_Ni_1.33_(PO_4_)_7_	1.33	12.7	1.33Ni-TCP	10.3135(6)	37.171(3)	3953.8(8)
Ca_8.92_Ni_1.58_(PO_4_)_7_	1.58	15	1.58Ni-TCP	10.3109(5)	37.168(8)	3951.5(1)
Ca_10.17_Co_0.33_(PO_4_)_7_	0.33	3.1	0.33Co-TCP	10.3965(6)	37.333(1)	4035.2(9)
Ca_10.0_Co_0.5_(PO_4_)_7_	0.5	4.8	0.5Co-TCP	10.3811(9)	37.262(6)	4015.7(1)
Ca_9.83_Co_0.67_(PO_4_)_7_	0.67	6.4	0.67Co-TCP	10.3723(3)	37.238(8)	4006.2(6)
Ca_9.5_Co(PO_4_)_7_	1.0	9.5	1.00Co-TCP	10.3429(2)	37.179(5)	3997.3(4)

**Table 3:** MTT assay results performed on aMSC grown for 24 h in the presence of 0.1 mg/mL TCP, Ni-TCP (0.33, 0.67, 1.0, 1.33, and 1.58), and Co-TCP (0.33, 0.5, 0.67, and 1.0). The reported values of OD600, % cell growth, and ±SD are the means of three experiments.

Sample	OD Media	Growth, %	SD
Cell control	0.151	100	0.021
TCP	0.151	100.24	0.008
0.33Ni-TCP	0.157	103.95	0.008
0.67Ni-TCP	0.154	101.90	0.009
1.00Ni-TCP	0.152	100.38	0.003
1.33Ni-TCP	0.151	100.11	0.005
1.58Ni-TCP	0.146	96.53	0.006
0.33Co-TCP	0.149	98.68	0.001
0.50Co-TCP	0.146	96.47	0.004
0.67Co-TCP	0.135	89.38	0.013
1.00Co-TCP	0.135	89.43	0.014

## Data Availability

The research data are available upon an official, reasonable request.

## Supplementary Materials

The following supporting information can be downloaded at: https://www.mdpi.com/article/10.3390/biomimetics9010014/s1, Figures S1–S9: Intensity profiles for the powder X-ray Rietveld refinement of Ca_10.5−*x*_Ni*_x_*(PO_4_)_7_ and Ca_10.5−*x*_Co*_x_*(PO_4_)_7_; Table S1: Main crystallographic and experimental data on Ca_10.5−*x*_Ni*_x_*(PO_4_)_7_ and Ca_10.5−*x*_Co*_x_*(PO_4_)_7_; Tables S2–S10: Atomic coordinates, displacement parameters (Å^2^), and site-occupancy factors (SOFs) in the structure of the synthesized samples.
