# Supplementary material for: Antimicrobial and Cell-Friendly Properties of Cobalt and Nickel-Doped Tricalcium Phosphate Ceramics
Source: Biomimetics (Basel). 2023 Dec 31;9(1):14. doi: 10.3390/biomimetics9010014 (PMC10813436; doi:10.3390/biomimetics9010014)
Supplement: Supplementary file 1 [file biomimetics-09-00014-s001.zip › biomimetics-2737296-supplementary.pdf]

Supporting information

Antimicrobial and cell friendly properties of cobalt and nickel-doped tricalcium phosphate ceramics

Dina V. Deyneko [1,2,*], Vladimir N. Lebedev [1], Katia Barbaro [3], Vladimir V. Titkov [1], Bogdan I. Lazoryak [1], Inna V. Fadeeva [4], Alevtina N. Gosteva [5], Irina L. Udyanskaya [6], Sergey M. Aksenov [2,7] and Julietta V. Rau [6,8,*]

[1] Chemistry Department, Lomonosov Moscow State University, Leninskie Gory 1, 119991 Moscow, Russia; vladimir.lebedev@chemistry.msu.ru (V.N.L.); vlatitkov@yandex.ru (V.V.T.); bilazoryak@gmail.com (B.I.L.)
[2] Laboratory of Arctic Mineralogy and Material Sciences, Kola Science Centre RAS, 14 Fersman Str., 184209 Apatity, Russia; aks.crys@gmail.com
[3] Istituto Zooprofilattico Sperimentale Lazio e Toscana "M. Aleandri", Via Appia Nuova 1411, 00178 Rome, Italy; katia.barbaro@izslt.it
[4] A.A. Baikov Institute of Metallurgy and Material Science, Russian Academy of Sciences, Leninsky Prospect 49, 119334 Moscow, Russia; fadeeva_inna@mail.ru
[5] Tananaev Institute of Chemistry, Kola Science Centre RAS, Akademgorodok 26A, 184209 Apatity, Russia; angosteva@list.ru
[6] Department of Analytical, Physical and Colloid Chemistry, Institute of Pharmacy, I.M. Sechenov First Moscow State Medical University, Trubetskaya 8, Build. 2, 119048 Moscow, Russia; udyanskaya_i_l@staff.sechenov.ru
[7] Geological Institute, Kola Science Centre, Russian Academy of Sciences, 14 Fersman Street, 184209 Apatity, Russia
[8] Istituto di Struttura della Materia, Consiglio Nazionale delle Ricerche (ISM-CNR), Via del Fosso del Cavaliere 100, 00133 Rome, Italy
* Correspondence: deynekomsu@gmail.com (D.V.D.); giulietta.rau@ism.cnr.it (J.V.R.)

Table S1. Main crystallographic and experimental data on $Ca_{10.5-x}Ni_x(PO_4)_7$ and $Ca_{10.5-x}Co_x(PO_4)_7$.

Sample	TCP	0.33Ni-TCP	0.67Ni-TCP	1.00Ni-TCP	1.33Ni-TCP	0.33Co-TCP	0.50Co-TCP	0.67Co-TCP	1.00Co-TCP
Temperature, K	293								
Crystal system, space group	Trigonal, *R*3*c*								
Radiation type	Cu *K*α								
Diffractometer	Rigaku SmartLab SE								
θ-Range	3.000- 80.000, step size (°) 0.02								
R_p	7.39	6.25	6.53	5.92	6.29	6.60	6.39	6.16	5.27
R_{wp}	10.21	8.08	8.22	7.68	8.41	8.74	8.75	8.22	7.29
R_{Bragg}	3.71	3.36	3.36	3.42	2.94	3.34	3.07	3.08	2.71
Goodness of fit (ChiQ)	2.75	2.40	2.45	2.24	2.86	2.62	2.85	2.67	2.69
Max./min. residual density ($e\times Å^{-3}$)	0.39/-0.29	0.52/-0.45	0.58/-0.64	1.76/-1.24	1.78/-1.35	0.59/-0.68	0.66/-1.17	0.58/-0.97	0.59/-1.17
No. of parameters	93	87	66	88	87	81	91	89	91

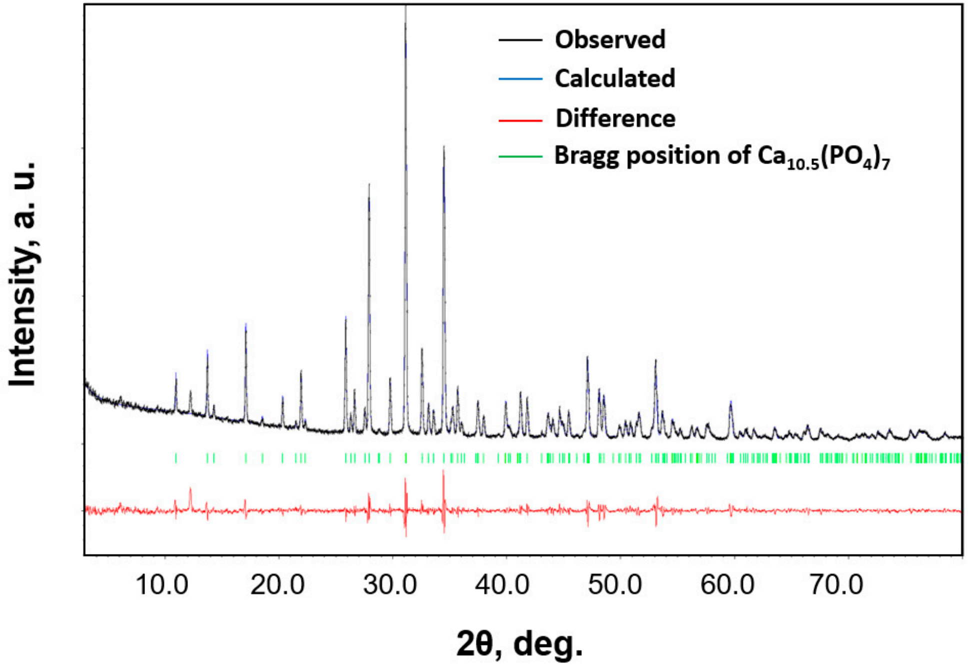

Figure S1 Intensity profiles for the powder X-ray Rietveld refinement of $Ca_{10.5}(PO_4)_7$. The observed and calculated profiles are represented in black and blue lines, respectively. The difference in the profile is plotted at the bottom (red line). Vertical bars indicate the positions of the Bragg reflections (green bars).

Table S2. Atomic coordinates, displacement parameters (Å^2) and site-occupancy factors (SOFs) in the structure of $Ca_{10.5}(PO_4)_7$.

Atom	Wyckoff site	*x*	*y*	*z*	U_{iso}, Å^2	SOF
M1	18b	0.7241(9)	0.8555(5)	0.1670(6)	0.0183(8)	$Ca_{1.0}$
M2	18b	0.6208(6)	0.8196(8)	-0.0324(1)	0.0171(1)	$Ca_{1.0}$
M3	18b	0.7279(3)	0.8506(4)	0.0617(1)	0.0268(1)	$Ca_{1.0}$
M4	6a	0	0	-0.0841(4)	0.0960(1)	$Ca_{0.5}$
M5	6a	0	0	0.7354(1)	0.0434(1)	$Ca_{1.0}$
P1	6a	0	0	-0.0003(2)	0.0185(7)	$P_{1.0}$
P2	18b	0.6889(4)	0.8694(7)	0.8700(4)	0.0183(1)	$P_{1.0}$
P3	18b	0.6509(6)	0.8400(6)	0.7681(5)	0.0208(1)	$P_{1.0}$
O1	6a	0.7305(3)	-0.0962(3)	-0.0952(6)	0.0083	$O_{1.0}$
O2	6a	0.7678(5)	0.7860(9)	0.8572(9)	0.0083	$O_{1.0}$
O3	18b	0.7278(4)	0.0099(2)	0.8441(7)	0.0083	$O_{1.0}$
O4	18b	0.5280(6)	0.7512(5)	0.8614(0)	0.0083	$O_{1.0}$
O5	18b	0.5975(0)	-0.0452(5)	0.7793(6)	0.0083	$O_{1.0}$
O6	6a	0.5716(3)	0.6867(5)	0.7862(2)	0.0083	$O_{1.0}$
O7	18b	0.0778(3)	0.8967(5)	0.7767(5)	0.0083	$O_{1.0}$
O8	18b	0.6309(5)	0.8307(0)	0.7272(8)	0.0083	$O_{1.0}$
O9	18b	0.0055(7)	0.8639(5)	-0.0208(7)	0.0083	$O_{1.0}$
O10	18b	0	0	0.0408(4)	0.0083	$O_{1.0}$

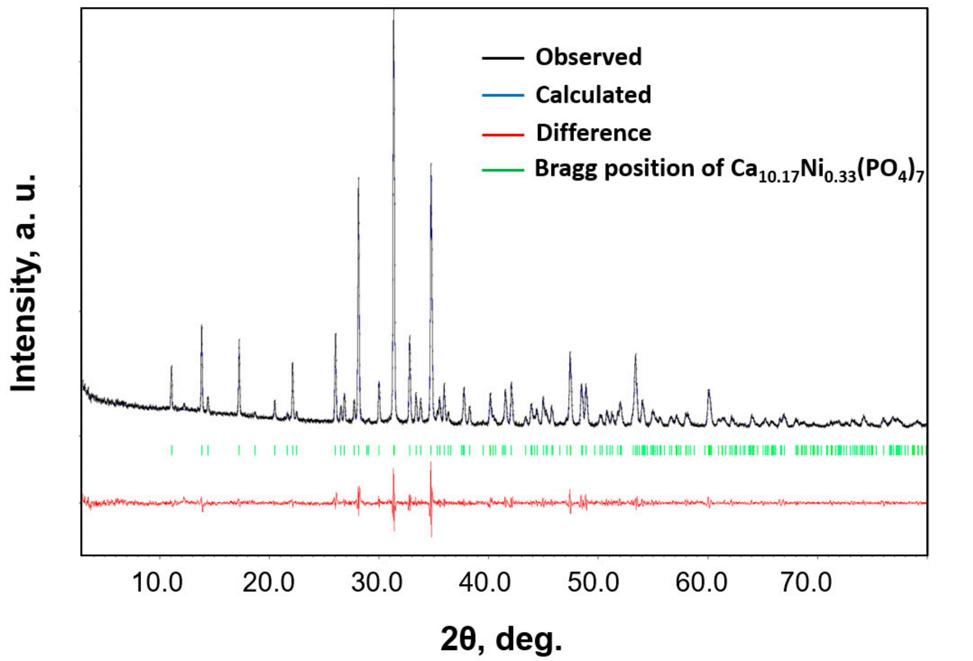

Figure S2. Intensity profiles for the powder X-ray Rietveld refinement of $Ca_{10.17}Ni_{0.33}(PO_4)_7$. The observed and calculated profiles are represented in black and blue lines, respectively. The difference in the profile is plotted at the bottom (red line). Vertical bars indicate the positions of the Bragg reflections (green bars).

Table S3. Atomic coordinates, displacement parameters (Å^2) and site-occupancy factors (SOFs) in the structure of $Ca_{10.17}Ni_{0.33}(PO_4)_7$.

Atom	Wyckoff site	*x*	*y*	*z*	U_{iso}, Å^2	SOF
M1	18b	0.7240(9)	0.8548(3)	0.1681(4)	0.0146(1)	$Ca_{1.0}$
M2	18b	0.6221(2)	0.8211(5)	-0.0320(1)	0.0316(1)	$Ca_{1.0}$
M3	18b	0.7261(4)	0.8488(7)	0.0619(1)	0.0142(2)	$Ca_{1.0}$
M4	6a	0	0	-0.0771(0)	0.0056(7)	$Ca_{0.5}$
M5	6a	0	0	0.7381(7)	0.0108(7)	$Ca_{0.67}$+$Ni_{0.33}$
P1	6a	0	0	0.0011(9)	0.0172(9)	$P_{1.0}$
P2	18b	0.6911(9)	0.8623(5)	0.8704(4)	0.0352(8)	$P_{1.0}$
P3	18b	0.6514(3)	0.8486(2)	0.7674(9)	0.0133(1)	$P_{1.0}$
O1	6a	0.7418(9)	-0.0911(1)	-0.0940(7)	0.0083	$O_{1.0}$
O2	6a	0.7582(0)	0.7658(2)	0.8584(5)	0.0083	$O_{1.0}$
O3	18b	0.7416(0)	0.0088(0)	0.8466(9)	0.0083	$O_{1.0}$
O4	18b	0.5225(7)	0.7585(9)	0.8651(1)	0.0083	$O_{1.0}$
O5	18b	0.6105(9)	-0.0358(9)	0.7835(7)	0.0083	$O_{1.0}$
O6	6a	0.5588(5)	0.6993(7)	0.7872(1)	0.0083	$O_{1.0}$
O7	18b	0.0763(2)	0.8975(3)	0.7742(8)	0.0083	$O_{1.0}$
O8	18b	0.6189(3)	0.8116(8)	0.7272(9)	0.0083	$O_{1.0}$
O9	18b	0.0013(4)	0.8629(3)	-0.0188(3)	0.0083	$O_{1.0}$
O10	18b	0	0	0.0425(6)	0.0083	$O_{1.0}$

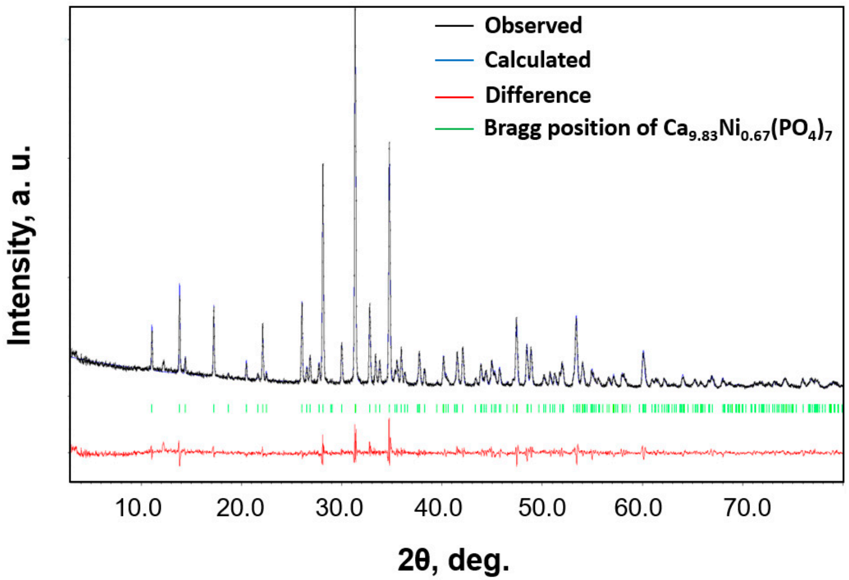

Figure S3. Intensity profiles for the powder X-ray Rietveld refinement of $Ca_{9.83}Ni_{0.67}(PO_4)_7$. The observed and calculated profiles are represented in black and blue lines, respectively. The difference in the profile is plotted at the bottom (red line). Vertical bars indicate the positions of the Bragg reflections (green bars).

Table S4. Atomic coordinates, displacement parameters (Å^2) and site-occupancy factors (SOFs) in the structure of $Ca_{9.83}Ni_{0.67}(PO_4)_7$.

Atom	Wyckoff site	*x*	*y*	*z*	U_{iso}, Å^2	SOF
M1	18b	0.7255(7)	0.8561(1)	0.1674(4)	0.0166(6)	$Ca_{1.0}$
M2	18b	0.6184(3)	0.8175(7)	-0.0326(1)	0.0226(8)	$Ca_{1.0}$
M3	18b	0.7257(3)	0.8488(9)	0.0615(1)	0.0196(9)	$Ca_{1.0}$
M4	6a	0	0	-0.0793(4)	0.0851(0)	$Ca_{0.5}$
M5	6a	0	0	0.7361(5)	0.0221(1)	$Ca_{0.33}$+$Ni_{0.67}$
P1	6a	0	0	-0.0007(3)	0.0328(9)	$P_{1.0}$
P2	18b	0.6900(0)	0.8625(0)	0.8705(2)	0.0088(7)	$P_{1.0}$
P3	18b	0.6546(6)	0.8500(4)	0.7667(7)	0.0083	$P_{1.0}$
O1	6a	0.7389(5)	-0.0861(1)	-0.0936(4)	0.0083	$O_{1.0}$
O2	6a	0.7587(1)	0.7674(5)	0.8584(7)	0.0083	$O_{1.0}$
O3	18b	0.7314(5)	-0.0021(8)	0.8453(9)	0.0083	$O_{1.0}$
O4	18b	0.5216(6)	0.7531(1)	0.8663(1)	0.0083	$O_{1.0}$
O5	18b	0.6018(2)	-0.0342(4)	0.7825(2)	0.0083	$O_{1.0}$
O6	6a	0.5656(3)	0.7005(5)	0.7866(9)	0.0083	$O_{1.0}$
O7	18b	0.0729(9)	0.8948(1)	0.7757(3)	0.0083	$O_{1.0}$
O8	18b	0.6190(8)	0.8135(1)	0.7266(5)	0.0083	$O_{1.0}$
O9	18b	-0.0016(1)	0.8546(6)	-0.0145(3)	0.0083	$O_{1.0}$
O10	18b	0	0	0.0406(4)	0.0083	$O_{1.0}$

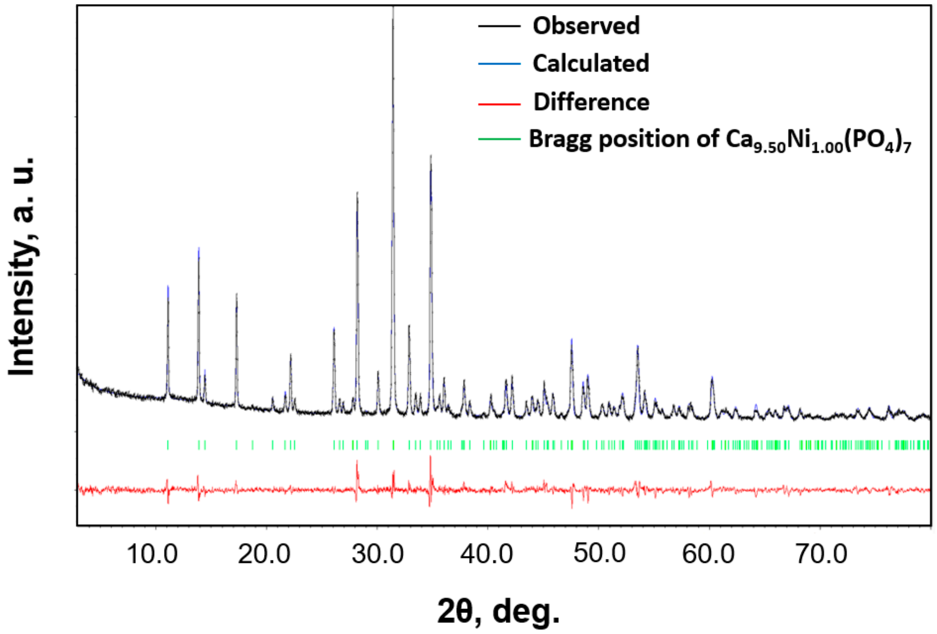

Figure S4. Intensity profiles for the powder X-ray Rietveld refinement of $Ca_{9.50}Ni_{1.00}(PO_4)_7$. The observed and calculated profiles are represented in black and blue lines, respectively. The difference in the profile is plotted at the bottom (red line). Vertical bars indicate the positions of the Bragg reflections (green bars).

Table S5. Atomic coordinates, displacement parameters (Å^2) and site-occupancy factors (SOFs) in the structure of $Ca_{9.50}Ni_{1.00}(PO_4)_7$.

Atom	Wyckoff site	*x*	*y*	*z*	U_{iso}, Å^2	SOF
M1	18b	0.7167(9)	0.8453(3)	0.1688(0)	0.0113(7)	$Ca_{1.0}$
M2	18b	0.6273(0)	0.8233(0)	-0.0338(9)	0.0213(3)	$Ca_{1.0}$
M3	18b	0.7225(3)	0.8523(8)	0.0608(2)	0.0253(9)	$Ca_{1.0}$
M4	6a	0	0	-0.0828(7)	0.0228(8)	$Ca_{0.5}$
M5	6a	0	0	0.7329(3)	0.0244(6)	$Ni_{1.00}$
P1	6a	0	0	0.0029(2)	0.0155(3)	$P_{1.0}$
P2	18b	0.6766(8)	0.8548(5)	0.8690(4)	0.0009(1)	$P_{1.0}$
P3	18b	0.6338(9)	0.8288(1)	0.7649(8)	0.0083	$P_{1.0}$
O1	6a	0.7450(1)	-0.0884(9)	-0.0893(4)	0.0083	$O_{1.0}$
O2	6a	0.7722(2)	0.8148(5)	0.8515(8)	0.0083	$O_{1.0}$
O3	18b	0.7206(0)	-0.0081(6)	0.8453(1)	0.0083	$O_{1.0}$
O4	18b	0.5141(2)	0.7461(4)	0.8683(5)	0.0083	$O_{1.0}$
O5	18b	0.6162(9)	-0.0359(5)	0.7738(1)	0.0083	$O_{1.0}$
O6	6a	0.5875(5)	0.6779(0)	0.7834(8)	0.0083	$O_{1.0}$
O7	18b	0.0753(8)	0.8900(5)	0.7729(7)	0.0083	$O_{1.0}$
O8	18b	0.6208(1)	0.8206(9)	0.7266(8)	0.0083	$O_{1.0}$
O9	18b	-0.0090(2)	0.8730(6)	-0.0256(7)	0.0083	$O_{1.0}$
O10	18b	0	0	0.0433(7)	0.0083	$O_{1.0}$

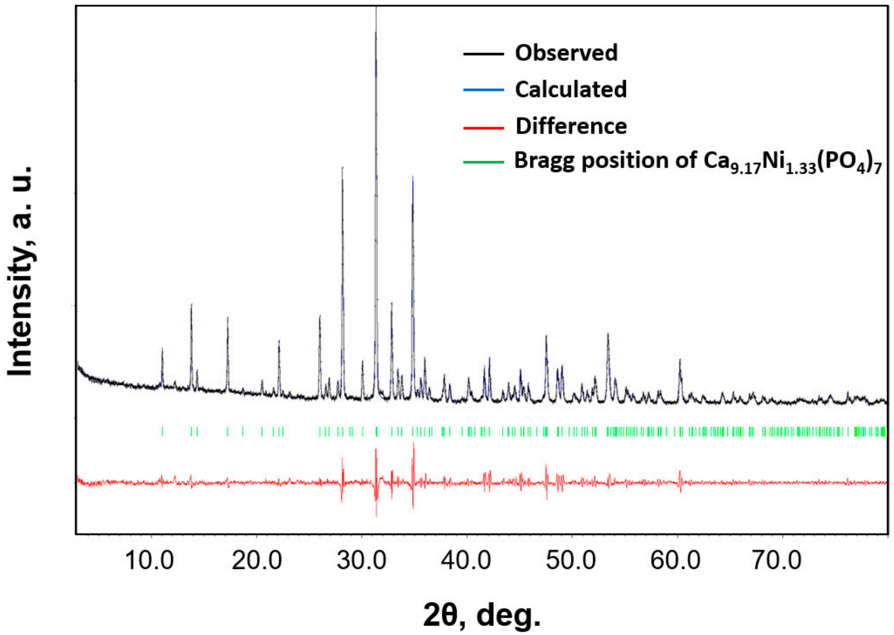

Figure S5. Intensity profiles for the powder X-ray Rietveld refinement of $Ca_{9.17}Ni_{1.33}(PO_4)_7$. The observed and calculated profiles are represented in black and blue lines, respectively. The difference in the profile is plotted at the bottom (red line). Vertical bars indicate the positions of the Bragg reflections (green bars).

Table S6. Atomic coordinates, displacement parameters (Å^2) and site-occupancy factors (SOFs) in the structure of $Ca_{9.17}Ni_{1.33}(PO_4)_7$.

Atom	Wyckoff site	*x*	*y*	*z*	U_{iso}, Å^2	SOF
M1	18b	0.7119(9)	0.8606(2)	0.1712(1)	0.0247(2)	Ca+Ni
M2	18b	0.6127(1)	0.8224(5)	-0.0309(9)	0.0167(5)	$Ca_{1.0}$
M3	18b	0.7237(6)	0.8459(8)	0.0635(7)	0.0270(3)	Ca+Ni
M4	6a	0	0	-0.0779(3)	0.0307(6)	$Ca_{0.5}$
M5	6a	0	0	0.7392(8)	0.0144(1)	$Ni_{1.00}$
P1	6a	0	0	0.0002(3)	0.0615(1)	$P_{1.0}$
P2	18b	0.6847(0)	0.8546(5)	0.8707(6)	0.0164(5)	$P_{1.0}$
P3	18b	0.6561(7)	0.8621(4)	0.7681(6)	0.0833(9)	$P_{1.0}$
O1	6a	0.7886(5)	-0.0415(6)	-0.0843(5)	0.0083	$O_{1.0}$
O2	6a	0.7475(3)	0.7576(8)	0.8558(8)	0.0083	$O_{1.0}$
O3	18b	0.7408(8)	-0.0314(5)	0.8518(4)	0.0083	$O_{1.0}$
O4	18b	0.5136(9)	0.7881(6)	0.8720(1)	0.0083	$O_{1.0}$
O5	18b	0.6078(4)	-0.0241(5)	0.7695(5)	0.0083	$O_{1.0}$
O6	6a	0.5613(4)	0.7048(1)	0.7847(1)	0.0083	$O_{1.0}$
O7	18b	0.0673(2)	0.8837(4)	0.7818(6)	0.0083	$O_{1.0}$
O8	18b	0.6205(9)	0.8223(0)	0.7277(0)	0.0083	$O_{1.0}$
O9	18b	-0.0255(1)	0.9040(2)	-0.0287(5)	0.0083	$O_{1.0}$
O10	18b	0	0	0.0416(9)	0.0083	$O_{1.0}$

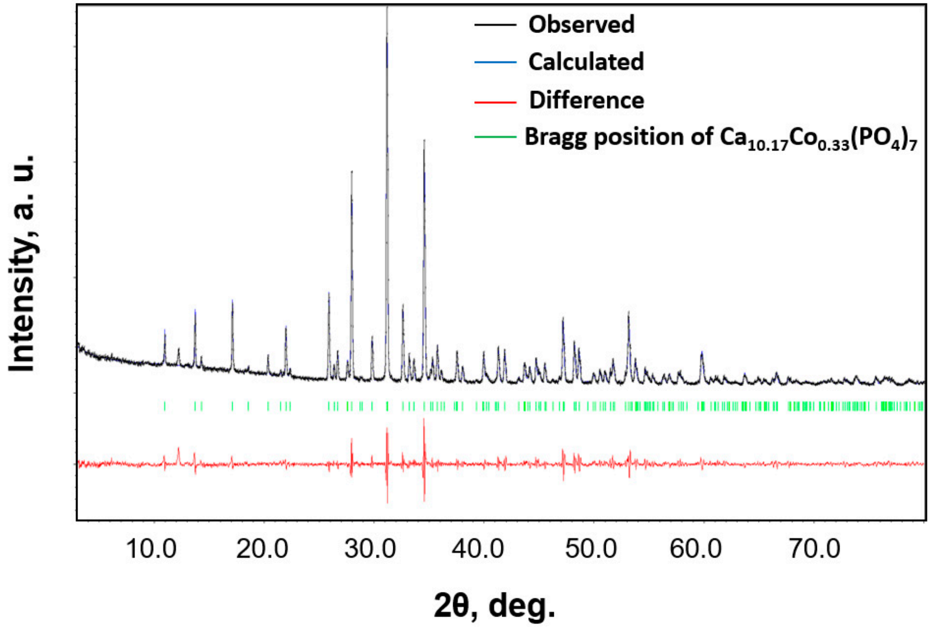

Figure S6. Intensity profiles for the powder X-ray Rietveld refinement of $Ca_{10.17}Co_{0.33}(PO_4)_7$. The observed and calculated profiles are represented in black and blue lines, respectively. The difference in the profile is plotted at the bottom (red line). Vertical bars indicate the positions of the Bragg reflections (green bars).

Table S7. Atomic coordinates, displacement parameters (Å^2) and site-occupancy factors (SOFs) in the structure of $Ca_{10.17}Co_{0.33}(PO_4)_7$.

Atom	Wyckoff site	*x*	*y*	*z*	U_{iso}, Å^2	SOF
M1	18b	0.7292(3)	0.8573(9)	0.1684(8)	0.0280(1)	$Ca_{1.0}$
M2	18b	0.6216(8)	0.8251(7)	-0.0318(8)	0.0091(1)	$Ca_{1.0}$
M3	18b	0.7259(6)	0.8571(0)	0.0624(7)	0.0596(2)	$Ca_{1.0}$
M4	6a	0	0	-0.0805(9)	0.0415(6)	$Ca_{0.5}$
M5	6a	0	0	0.7374(2)	0.0387(1)	$Ca_{0.67}$+$Co_{0.33}$
P1	6a	0	0	-0.0039(4)	0.0563(0)	$P_{1.0}$
P2	18b	0.6978(5)	0.8673(2)	0.8701(0)	0.0437(5)	$P_{1.0}$
P3	18b	0.6587(6)	0.8479(7)	0.7664(6)	0.0063(8)	$P_{1.0}$
O1	6a	0.7203(6)	-0.0952(4)	-0.0859(6)	0.0083(0)	$O_{1.0}$
O2	6a	0.7694(8)	0.7726(0)	0.8609(6)	0.0083(0)	$O_{1.0}$
O3	18b	0.7375(0)	0.0079(3)	0.8514(4)	0.0083(0)	$O_{1.0}$
O4	18b	0.5275(3)	0.7725(4)	0.8669(2)	0.0083(0)	$O_{1.0}$
O5	18b	0.5975(7)	-0.0438(6)	0.7852(1)	0.0083(0)	$O_{1.0}$
O6	6a	0.5759(6)	0.7216(1)	0.7901(8)	0.0083(0)	$O_{1.0}$
O7	18b	0.0826(5)	0.9060(1)	0.7788(2)	0.0083(0)	$O_{1.0}$
O8	18b	0.6091(1)	0.7985(2)	0.7330(1)	0.0083(0)	$O_{1.0}$
O9	18b	-0.0140(6)	0.8438(0)	-0.0216(9)	0.0083(0)	$O_{1.0}$
O10	18b	0	0	0.0373(1)	0.0083(0)	$O_{1.0}$

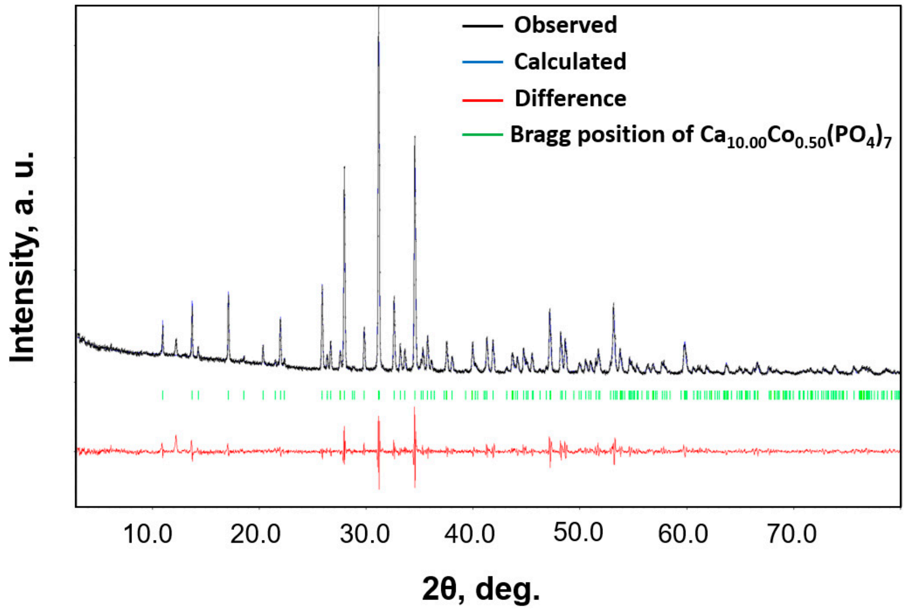

Figure S7. Intensity profiles for the powder X-ray Rietveld refinement of $Ca_{10.00}Co_{0.50}(PO_4)_7$. The observed and calculated profiles are represented in black and blue lines, respectively. The difference in the profile is plotted at the bottom (red line). Vertical bars indicate the positions of the Bragg reflections (green bars).

Table S8. Atomic coordinates, displacement parameters (Å^2) and site-occupancy factors (SOFs) in the structure of $Ca_{10.00}Co_{0.50}(PO_4)_7$.

Atom	Wyckoff site	*x*	*y*	*z*	U_{iso}, Å^2	SOF
M1	18b	0.7219(7)	0.8550(9)	0.1666(3)	0.0568(7)	$Ca_{1.0}$
M2	18b	0.6153(9)	0.8194(9)	-0.0335(1)	0.0087(3)	$Ca_{1.0}$
M3	18b	0.7272(1)	0.8519(7)	0.0611(7)	0.0489(9)	$Ca_{1.0}$
M4	6a	0	0	-0.0796(3)	0.0111(5)	$Ca_{0.5}$
M5	6a	0	0	0.7367(4)	0.0212(9)	$Ca_{0.50}$+$Co_{0.50}$
P1	6a	0	0	-0.0001(7)	0.0121(1)	$P_{1.0}$
P2	18b	0.6959(4)	0.8627(1)	0.8693(9)	0.0459(2)	$P_{1.0}$
P3	18b	0.6575(8)	0.8515(2)	0.7673(7)	0.0059(1)	$P_{1.0}$
O1	6a	0.7288(4)	-0.0916(6)	-0.0930(1)	0.0083	$O_{1.0}$
O2	6a	0.7629(5)	0.7657(9)	0.8580(2)	0.0083	$O_{1.0}$
O3	18b	0.7417(9)	0.0084(8)	0.8467(6)	0.0083	$O_{1.0}$
O4	18b	0.5262(1)	0.7653(1)	0.8647(2)	0.0083	$O_{1.0}$
O5	18b	0.6158(3)	-0.0394(3)	0.7834(8)	0.0083	$O_{1.0}$
O6	6a	0.5657(1)	0.6991(8)	0.7857(7)	0.0083	$O_{1.0}$
O7	18b	0.0750(3)	0.8958(6)	0.7764(3)	0.0083	$O_{1.0}$
O8	18b	0.6221(1)	0.8162(5)	0.7272(3)	0.0083	$O_{1.0}$
O9	18b	-0.0049(3)	0.8598(3)	-0.0195(5)	0.0083	$O_{1.0}$
O10	18b	0	0	0.0411(6)	0.0083	$O_{1.0}$

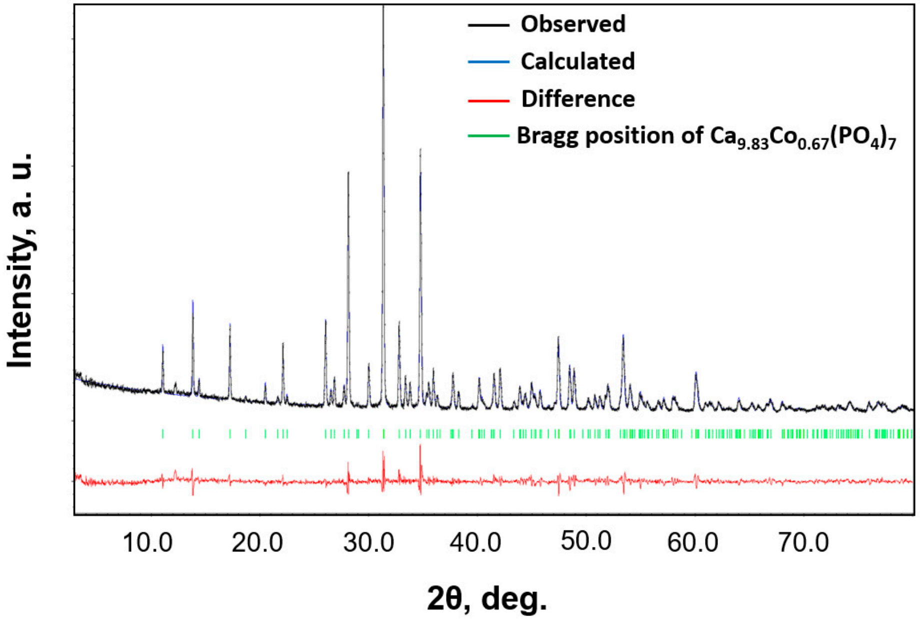

Figure S8. Intensity profiles for the powder X-ray Rietveld refinement of $Ca_{9.83}Co_{0.67}(PO_4)_7$. The observed and calculated profiles are represented in black and blue lines, respectively. The difference in the profile is plotted at the bottom (red line). Vertical bars indicate the positions of the Bragg reflections (green bars).

Table S9. Atomic coordinates, displacement parameters (Å^2) and site-occupancy factors (SOFs) in the structure of $Ca_{9.83}Co_{0.67}(PO_4)_7$.

Atom	Wyckoff site	*x*	*y*	*z*	U_{iso}, Å^2	SOF
M1	18b	0.7262(5)	0.8556(8)	0.1670(8)	0.0509(1)	$Ca_{1.0}$
M2	18b	0.6182(8)	0.8201(9)	-0.0330(0)	0.0028(0)	$Ca_{1.0}$
M3	18b	0.7255(5)	0.8539(1)	0.0607(4)	0.0481(5)	$Ca_{1.0}$
M4	6a	0	0	-0.0798(7)	0.0107(9)	$Ca_{0.5}$
M5	6a	0	0	0.7371(2)	0.0196(3)	$Ca_{0.33}$+$Co_{0.67}$
P1	6a	0	0	0.0010(1)	0.0104(5)	$P_{1.0}$
P2	18b	0.6971(5)	0.8681(6)	0.8701(9)	0.0290(3)	$P_{1.0}$
P3	18b	0.6599(4)	0.8513(3)	0.7669(9)	0.0015(5)	$P_{1.0}$
O1	6a	0.7238(4)	-0.0918(7)	-0.0897(1)	0.0083	$O_{1.0}$
O2	6a	0.7670(8)	0.7751(4)	0.8577(5)	0.0083	$O_{1.0}$
O3	18b	0.7306(4)	0.0056(3)	0.8479(4)	0.0083	$O_{1.0}$
O4	18b	0.5289(1)	0.7667(5)	0.8638(0)	0.0083	$O_{1.0}$
O5	18b	0.6088(6)	-0.0409(6)	0.7823(7)	0.0083	$O_{1.0}$
O6	6a	0.5751(3)	0.7038(5)	0.7878(3)	0.0083	$O_{1.0}$
O7	18b	0.0788(1)	0.9048(1)	0.7762(7)	0.0083	$O_{1.0}$
O8	18b	0.6159(9)	0.8148(2)	0.7272(4)	0.0083	$O_{1.0}$
O9	18b	-0.0105(7)	0.8541(5)	-0.0217(7)	0.0083	$O_{1.0}$
O10	18b	0	0	0.0423(7)	0.0083	$O_{1.0}$

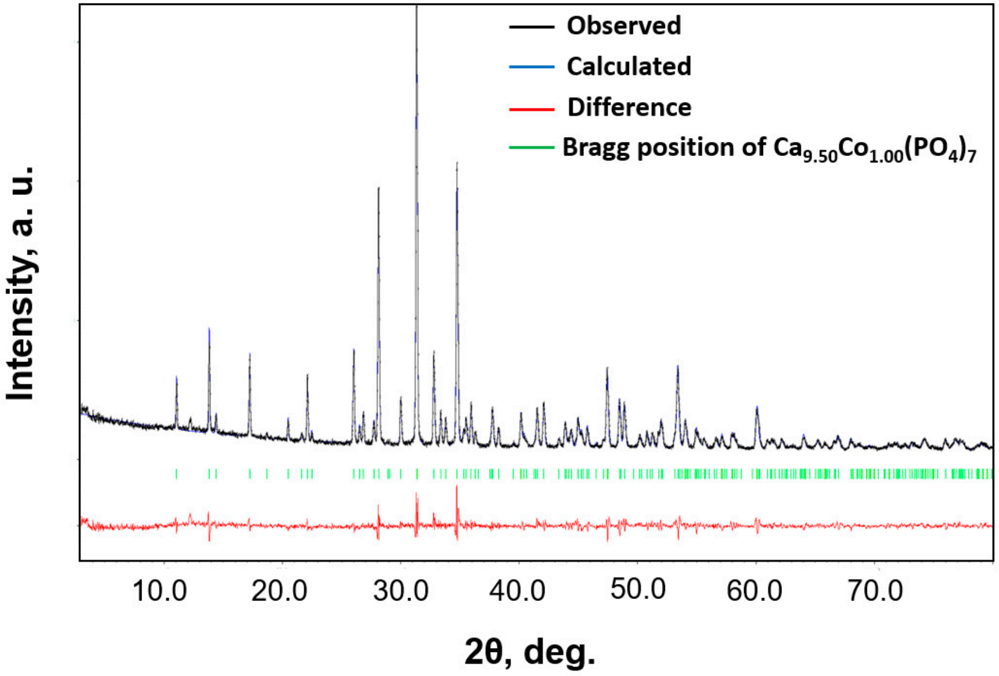

Figure S9. Intensity profiles for the powder X-ray Rietveld refinement of $Ca_{9.50}Co_{1.00}(PO_4)_7$. The observed and calculated profiles are represented in black and blue lines, respectively. The difference in the profile is plotted at the bottom (red line). Vertical bars indicate the positions of the Bragg reflections (green bars).

Table S10. Atomic coordinates, displacement parameters (Å^2) and site-occupancy factors (SOFs) in the structure of $Ca_{9.50}Co_{1.00}(PO_4)_7$.

Atom	Wyckoff site	*x*	*y*	*z*	U_{iso}, Å^2	SOF
M1	18b	0.7293(5)	0.8580(6)	0.1681(8)	0.0413(4)	$Ca_{1.0}$
M2	18b	0.6197(9)	0.8219(7)	-0.0324(6)	0.0066(3)	$Ca_{1.0}$
M3	18b	0.7273(6)	0.8553(1)	0.0620(9)	0.0486(8)	$Ca_{1.0}$
M4	6a	0	0	-0.0766(2)	0.0716(4)	$Ca_{0.5}$
M5	6a	0	0	0.7387(7)	0.0186(6)	$Co_{1.00}$
P1	6a	0	0	0.0012(1)	0.0213(8)	$P_{1.0}$
P2	18b	0.6992(3)	0.8624(7)	0.8720(4)	0.0507(1)	$P_{1.0}$
P3	18b	0.6586(0)	0.8525(0)	0.7676(8)	0.0095(6)	$P_{1.0}$
O1	6a	0.7267(3)	-0.0867(3)	-0.0887(5)	0.0083	$O_{1.0}$
O2	6a	0.7675(1)	0.7677(6)	0.8593(3)	0.0083	$O_{1.0}$
O3	18b	0.7407(9)	0.0027(2)	0.8488(7)	0.0083	$O_{1.0}$
O4	18b	0.5288(2)	0.7804(6)	0.8664(6)	0.0083	$O_{1.0}$
O5	18b	0.6143(6)	-0.0364(2)	0.7835(5)	0.0083	$O_{1.0}$
O6	6a	0.5701(3)	0.7083(7)	0.7898(4)	0.0083	$O_{1.0}$
O7	18b	0.0770(3)	0.9076(8)	0.7749(6)	0.0083	$O_{1.0}$
O8	18b	0.6109(8)	0.8016(4)	0.7285(5)	0.0083	$O_{1.0}$
O9	18b	-0.0130(0)	0.8502(6)	-0.0200(9)	0.0083	$O_{1.0}$
O10	18b	0	0	0.0426(8)	0.0083	$O_{1.0}$
